# Robust and ultra-stable nanohesive-based solid-like slippery coating under dynamic blood flow environment for durable prevention of thrombosis and biofouling

**DOI:** 10.1016/j.mtbio.2025.102449

**Published:** 2025-10-21

**Authors:** Shu Zhang, Jihua Zou, Yupeng Xiao, Xiaoying Qiu, Yao Shen, Yijin Zhao, Tao Fan, Manxu Zheng, Guozhi Huang, Qing Zeng, Chengduan Yang

**Affiliations:** a Center of Rehabilitation Medicine, Zhujiang Hospital, School of Rehabilitation Sciences, Southern Medical University, Guangzhou, China; b Faculty of Health and Social Sciences, Hong Kong Polytechnic University, Kowloon, Hong Kong Special Administrative Region of China

**Keywords:** “Solid-like” slippery coating (SSC), Nanohesive-based coating, Blood-contacting biomedical devices, Anti-biofouling and anti-thrombotic, Robust and ultra-stable resistance

## Abstract

Biomedical devices face thrombosis and infection risks due to nonspecific bioadhesion. Although liquid-infused surfaces (LIS) exhibit anti-biofouling potential, lubricant loss under blood flow limits their utility. An innovative “solid-like” slippery coating (SSC) addresses this via amino-functionalized SiO_2_ nanoparticles anchoring carboxy terminated silicone oil within an epoxy resin matrix. The collective effects of electrostatic interactions, hydrogen bonds and van der Waals forces between nanoparticles and silicone oil, in combination with epoxy resin encapsulation and dense microstructures form by the cross-linked nanoparticles, ensure lubricant retention and mechanical stability. Benefiting from the “slippery” properties, SSC exhibits exceptional resistance to various aqueous-based liquids, proteins, bacteria, cells, and platelets adhesion. Under conditions of low shear rate (250 s^−1^, 30 days) or high shear rate (1750 s^−1^, 7 days), SSC modified medical catheters maintain outstanding anti-fouling (>71 %) and anti-thrombotic (>67 %) capabilities, achieving ultra-stable anti-fouling performance under dynamic blood flow conditions. *In vivo* arteriovenous shunt and implanted experiments demonstrated that SSC effectively prevents blood clot, reduces inflammation, and avoids organ damage, with outstanding biocompatibility. The technology provides a durable solution for blood-contacting biomedical devices through synergistic physical anchoring and chemical bonding mechanisms, enabling long-term infection resistance and thrombus prevention in dynamic fluid environments. The simple fabrication method enhances clinical translation prospects for medical implants requiring stable biological interface performance.

## Graphical abstract

**Figure:**
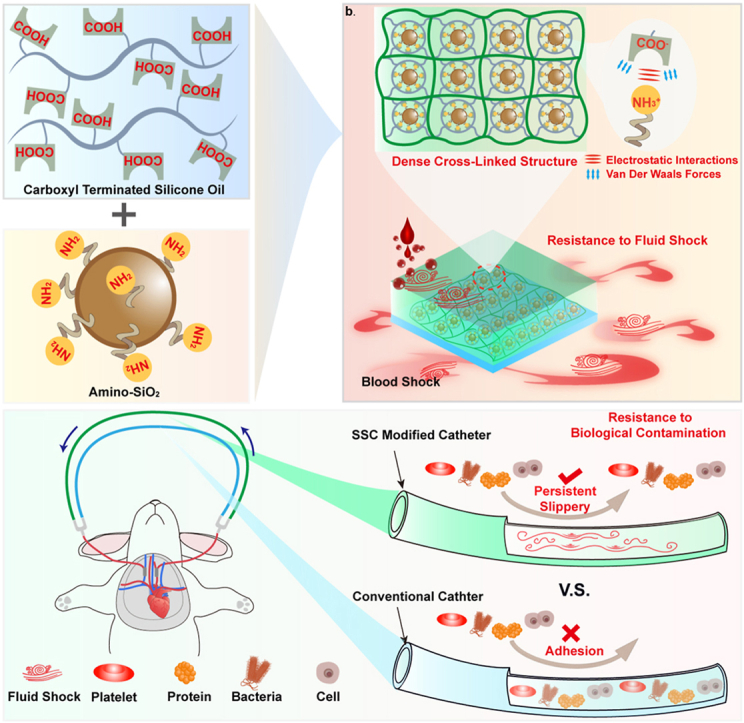

## 1 Introduction

Blood-contacting biomedical devices (e.g., cardiac pacemaker, vena cava filter, hemodialysis device, central venous catheter, urinary catheter and continuous blood glucose monitoring system) have been widely used for continuous drug administration and release, cancer treatment, internal surgery and other clinical treatments [[1], [2], [3], [4], [5]]. However, complications such as infection and thrombosis remain major challenges in the clinical application of blood-contacting biomedical devices. In past decades, anticoagulants and antibiotics were commonly used to alleviate clots and infections [[6], [7], [8]]. Unfortunately, systemic use of anticoagulants and antibiotics can easily cause adverse reactions such as heparin-induced thrombocytopenia (HIT), bleeding risk and antibiotic resistance [[9], [10], [11], [12]]. In fact, thrombosis is mainly caused by the adsorption and aggregation of nonspecific proteins, cells and platelets on the surface of the catheters, while infection is mainly related to the adsorption and colonization of bacteria (such as *E. coli* and *S*. *aureus*) in the catheters [[13], [14], [15], [16]]. Therefore, there is an urgent demand in clinical practices to develop a strategy of inhibiting biofouling, namely reducing proteins, cells, bacteria and platelets to colonizing surfaces of blood-contacting devices to effectively inhibit thrombosis and infection.

At present, effective inhibition of bioadhesion can be achieved by using non-drug delivery strategies, such as surface modification and reducing the interaction between biological substances and surfaces. So far, the surface modification strategies developed mainly include superhydrophilic, superhydrophobic and slippery strategy surface modifications [[17], [18], [19], [20]]. Among them, “slippery” modifications, especially omniphobic liquid-infused surfaces modifications (LIS or SLIPS), have been widely used to inhibit bioadhesion and thrombosis due to their advantages of biosafety, chemical stability and exceptional “slippery” properties [21]. For example, Yin's group created a fully omniphobic liquid-infused layer on the surface of a medical device with a fluorinated silane-functionalized surface that used interactions between fluorine groups to immobilize a perfluorocarbon lubricant, effectively inhibiting bacterial infection and blood clotting [22]. Unfortunately, these lubricant layers are typically anchored using porous, nanothorns, or simple physical adsorption only by van der Waals forces, resulting in low stability under dynamic fluid flow conditions [23]. For long-term blood-contacting equipments, the fluid flowing lubricant is constantly lost under the influence of shear rate, leading to functional failure [24,25]. Furthermore, current micro/nanoscale surface techniques, (e.g., lithography, laser ablation, and electrospinning) struggle with non-planar substrates (e.g., medical catheters) due to shape incompatibility. Such geometric constraints both hinder scalable production and trigger functional failure [26,27]. Although there is a high demand for functional surface modification techniques that offer durable, robust biofouling inhibition and biosafety, achieving these targets remains challenging.

Recent studies have found that “solid-like” slippery coating (SSC) developed by embedding oil-storage nanoparticles on smooth polymer surfaces (such as epoxy resins), which have exhibited exceptional “slippery” property for various aqueous-based liquids with low to ultra-high viscosities [[28], [29], [30]]. Based on these, whether relying on the SSC to achieve long-term inhibition of biological contamination and thrombosis in the blood flow environment still has the following problems: 1) The applications of SSC surface currently developed are still limited to the macro level (such as anti-liquid and anti-icing) [[28], [29], [30]], and whether it can inhibit the adhesion of biological substances such as proteins, bacteria, cells and platelets at the micro level and its mechanism of action have not been explored. 2) The key to achieve long-term anti-fouling and anti-coagulation based on the “slippery” strategy lies in how to fix the lubricant efficiently. It has been reported that surface-activated nanoparticles can act as adhesive to tightly adhere matching hydrogel to solid substrates through electrostatic interactions [31]. The Song's group demonstrated that nanoparticles play a crucial role in the stability of SSC, but the mechanism of the long-term action mechanism is not yet clear [28]. Whether further improvements can be made to SSC to achieve long-term stability of modified blood-contacting device surfaces in the blood flow environment remains to be seen. 3) Whether the SSC can be safely used in blood-contacting biomedical devices such as central venous catheters has not yet been revealed.

**Fig. 1:**
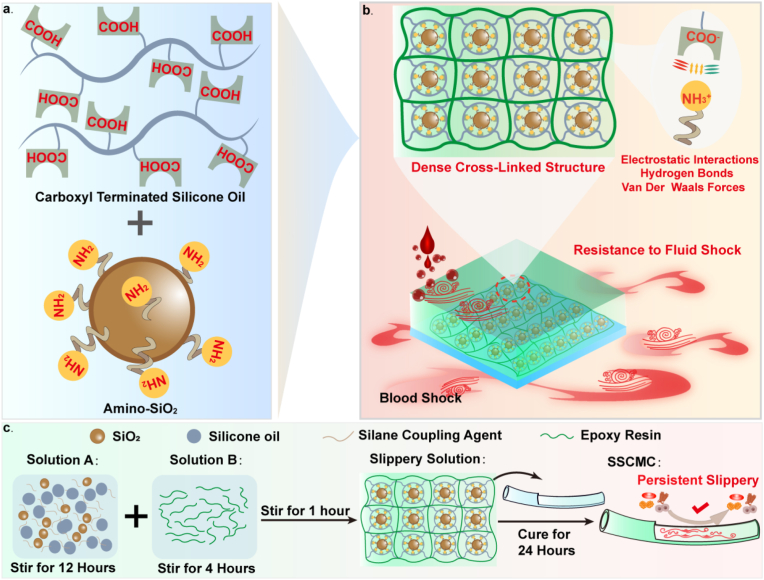
**Preparation of SSCMs.** a) Schematic diagram of -COOH terminated silicone oil and amino-functionalized SiO_2_ nanoparticles (amino-SiO_2_). b) The robust and ultra-stable resistance to fluid shock (blood) properties of the nanohesive-based SSC are mainly due to electrostatic interactions and dense structure. c) Schematic diagram of the fabrication procedure of SSCMC.

Inspired by the “anti-biofouling mechanism of slippery” and the “solid-like” slippery coating strategy, we developed an innovative nanohesive-based “solid-like” slippery coating by adjusting the composition of nanoparticles and silicone oil, using amino-functionalized SiO_2_ nanoparticles as adhesive to capture carboxyl (-COOH) terminated silicone oil and embed epoxy resin. The medical catheter was modified by atomizing spraying to provide persistent and robust resistance to thrombosis and biological contamination (Fig. 1a and b). The advantages and highlights of the nanohesive-based “solid-like” slippery coating are: 1) with exceptional “slippery” characteristics, the adhesion energy of the modified surface is significantly reduced, and the adhesion of proteins, bacteria, cells, platelets and thrombosis can be inhibited at the microscopic level, showing exceptional advantages against biological adhesion. 2) The innovative “solid-like” slippery coating utilizes the collective effect of molecular interactions such as the strong electrostatic interactions between particles and silicone oil, in conjunction with epoxy resin encapsulation, to achieve effective fixation of silicone oil. At the same time, the dense microstructure formed by the crosslinked nanoparticles can significantly improve the mechanical strength of the coating. The synergistic effect of these mechanisms facilitates the SSC to achieve long-term stability under fluid conditions. The innovative SSC modified catheter (SSCMC) showed exceptional resistance (>71 %) with low shear rate (∼250 s^−1^, for 30 days) or high shear rate (∼1750 s^−1^, for 7 days) blood flow environments. However, the LIS surface was subjected to blood flow impact for 7 days and had almost completely failed. To the best of our knowledge, this represents the most robust coating developed to date employing a “slippery” strategy, demonstrating prolonged inhibition of biocontamination and thrombosis in blood-contacting biomedical environments. 3) At the cellular and tissue levels, SSCMC showed outstanding biocompatibility, which does not affect protein and cell activity, and did not cause inflammation or organ/tissue damage *in vitro* blood circulation and *in vivo*. We provide a unique strategy to functionalize surfaces with biological safety, slippery *anti*-bioadhesion and ultra-stable durability, and will contribute to the development of implantable biomedical devices with robust and durable inhibition of biofouling and thrombosis. Moreover, it provides a new paradigm for the application of solid coating in the new field of biomedical antifouling resistance.

## 2 Results and discussion

### 2.1 Preparation and characterization of SSC-COOH modified surfaces (SSCMs)

**Fig. 2:**
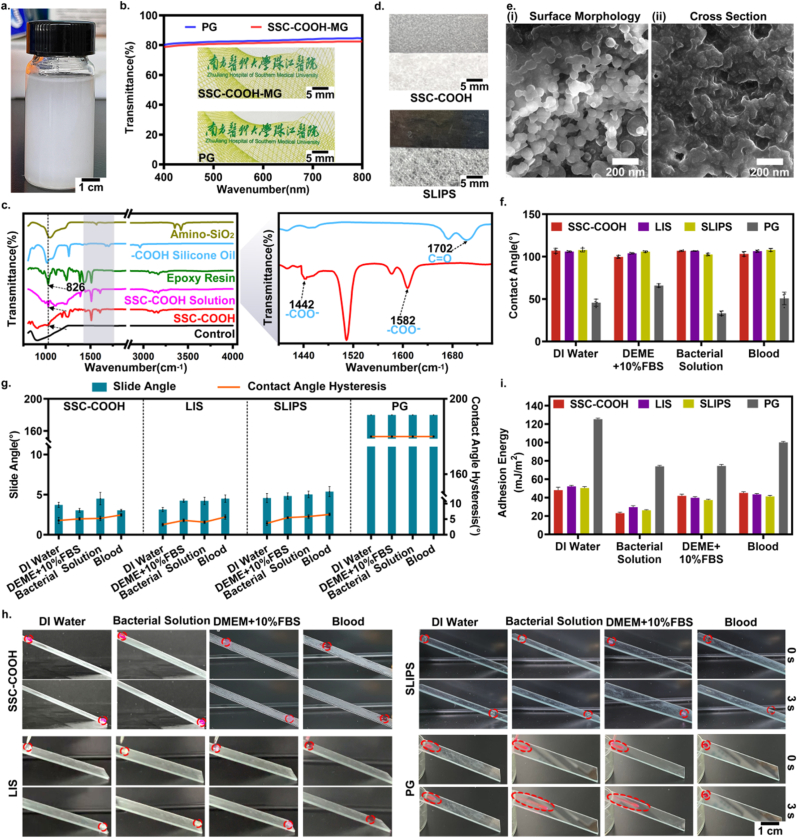
**Characterization of SSCMs.** a) The prepared coating solution was translucent. b) Transparency test. There was no obvious difference in transparency between SSC-COOH and PG, and the covered logo and letters were still clearly visible. c) Results of the FTIR spectrum analysis of amino-SiO_2_, -COOH terminated silicone oil, epoxy resin, SSC-COOH solution, SSC-COOH surface and control group (PC) surface. d) After the oil test paper was covered on the cured coating surface and showed a dry state to judge whether the silicone oil was fixed, while the image of a SLIPS covered with an oil test paper. It can be seen that the oil test paper was seriously and completely wetted by the flowing lubricant. e) SEM images of (i) surface morphology and (ii) cross section framework of SSC-COOH. f) Statistics of the CA of the four surfaces for four different types of liquid droplets, including SSC-COOH, LIS, SLIPS and PG. g) Statistics of the SA and CAH of the above four surfaces for four different types of liquid droplets (10 μl), where 180° indicates that the droplets in the corresponding surfaces showed diffusion and wetting phenomena, cannot be measured the specific value. h) Sliding of different liquid droplets (10 μl) on the glass surface before and after modification in 3 s. i) The adhesion energy of different surfaces to the DI water, bacterial solution, DEME +10 %FBS and blood were calculated and the resulting statistics were obtained. Error bar represents the mean ± SD. N = 4, averaged.

The fabrication procedures of the SSCMC were illustrated in Fig. 1c and S1. It was mainly completed through a two-step process of chemical reaction and spray. Firstly, the “solid-like” slippery solution was prepared through a series of chemical reactions including hydrolysis, condensation, cross-linking. Briefly, silane coupling agent was first hydrolyzed, and then condensed with the original nanoparticles to form amino-functionalized SiO_2_ surfaces. Based on the process, amination nanoparticles exhibited different degrees of affinity to a variety of terminated silicone oil (such as -COOH terminated, -OH terminated and -OCH_3_ terminated), enabling it to stable or unstable capture and store on the silica nanoparticles surface. The nanoparticles were cross-linked with the epoxy resin so that the oil-storing silica nanoparticles were embedded into the interior and the surface of the epoxy resin to obtain a translucent SSC solution (Fig. 2a). Secondly, the solution was uniformly sprayed on the entire surface of the catheters and spontaneously cured at room temperature to form SSC surface (SSC-COOH, SSC-OH, SSC-OCH_3_).

The composition and structure of SSC-COOH were characterized using light transmittance test, Fourier transform infrared (FTIR) spectroscopy, oil test paper test, scanning electron microscopy (SEM) and X-ray photoelectron spectroscopy (XPS). In the light transmittance test, glass was used as a substitute for medical catheter, representing SSC constructed with -COOH terminated silicone oil (SSC-COOH). Similar to the unmodified primitive glass (PG), the SSC-COOH exhibited high optical transparency (Fig. 2b), and the signs and letters below the SSC-COOH were also very clear, with no visual difference. The transmission spectra showed that in the visible region from 400 to 800 nm, the transmittance of the SSC-COOH (82.4 %) was comparable to the PG (84.7 %). However, as the thickness increases, the transparency will also decrease significantly (Fig. S2). These results indicated that the coating formed by the appropriate spray thickness has not significantly affect the appearance of the catheter, which has potential applications for implantable optical materials such as artificial crystals. To confirm the formation mechanism of the “solid-like” slippery coating, FTIR was first used to detect the characteristic peaks/bonding types of surface functional groups, including the characteristic peak changes of each component forming the “solid-like” slippery coating, SSC-COOH solution, and SSC-COOH-modified surface. As shown in Fig. 2c, compared with -COOH terminated silicone oil, the stretching vibration absorption peak was strengthened at 1442, 1582 cm^−1^, while the stretching vibration absorption peak of SSC-COOH disappeared at 1702 cm^−1^. It was attributed to the formation of strong electrostatic interactions in the SSC-COOH system [32]. Furthermore, we found that the characteristic peaks of epoxy groups in epoxy resin decreased by two times. Firstly, compared with epoxy resin, the epoxy characteristic peak (C-O-C, 826 cm^−1^) of the SSC-COOH solution decreased, which might be due to the covalent cross-linking of oil-trapping nanoparticles with epoxy resin. Secondly, after modifying the surface of the catheter, the epoxy characteristic peak further decreased, indicating that the SSC-COOH might have adhered to the surface of the catheter through covalent adhesion by relying on epoxy resin as the bonding layer. In addition, the oil test paper test results showed that the oil test paper remained dry and clean after covering the SSC-COOH modified surface (Fig. 2d), indicating that there was no residual lubricant on the surface, which contrasts with typical fully lubricant-infused porous surface (SLIPS) coating. It is worth noting that before the epoxy resin was added, namely SSC solution A, the oil test paper was wetted partially (Fig. S3), which means that the oil-trapping particles not cross-linked by the epoxy resin contain free lubricating silicone oil. These results indicate that after the nanoparticles strongly capture silicone oil molecules through electrostatic interactions, then capture the liquid flowing oil through van der Waals forces [24]. With the addition of epoxy resin, the nanoparticles that capture silicone oil were embedded into the surface and interior of the epoxy resin, and a dense structure was formed, thereby inhibiting the macroscopic migration and exudation of silicone oil. As shown in Fig. 2e, SEM images showed that the presence of nanoparticles that cross-link to each other to form a dense microstructure, which creates conditions for improving the mechanical stability (resistance to fluid shock) of SSC-COOH [33]. In addition, X-ray photoelectron spectroscopy (XPS) was used to analyze the elemental composition of SSCMC (including SSC-COOH, SSC-OH, SSC-OCH_3_). The Si and N group characteristic peaks of SSC surface at 102 ev (Si 2p peak), 153 ev (Si 2s peak) and 398 ev (N 1s peak) indicated that SSC polymer was successfully coated on the surface of catheters. No significant Si group and N group were observed on the surface of primitive catheter (PC) before coating (Fig. S4).

Considering the wetting and slippery properties as important indicators for evaluating the anti-biofouling performance, the wetting properties of SSC-COOH surfaces were characterized by measuring static contact angles (CA) of various aqueous-based liquids (deionized (DI) water, dulbecco's modified eagle medium containing 10 % fetal bovine serum (DMEM + 10 % FBS), the bacterial solution at a concentration of 1 × (10^9^-10^10^)/ml in broth and whole blood). Unmodified PG surfaces, typical omniphobic LIS and SLIPS surfaces were used as control groups, and the CA measurements were quantitatively analyzed. As shown in Fig. 2f, compared to the PG surfaces, the CAs of SSC-COOH with different droplets increased significantly, with CA reaching approximately 107° for DI water, 100° for DMEM +10 % FBS, 107° for bacterial solution, and 103° for whole blood. The wetting effect is comparable to LIS/SLIPS, but all no shown superhydrophobic state (CA < 150°). These results are close to our previous PDMS graft-forming liquid-like surface [34,35], suggesting that the increase in CA may be caused by the hydrophobicity of the silicone oil molecular backbone on the coated surface [36,37].

Further, the sliding performance of the SSC-COOH modified surface against different liquids (DI water, DMEM + 10 % FBS, bacterial solution and whole blood) were evaluated by slide angles (SA), contact angle hysteresis (CAH, CAH = *θ*_adv_ - *θ*_rec_) and dynamic sliding ability. As shown in Fig. 2g, similar to LIS and SLIPS, SSC showed very small SA and CAH for all liquids, all within 10°. Which suggested that the various aqueous-based droplets are highly prone to slipping from the SSC-COOH surface, in sharp contrast to the PG surface that was almost completely unable to slide. Further dynamic sliding images (Fig. 2h) showed that above various aqueous-based droplets can easily slide from the LIS, SLIPS and SSC-COOH surfaces (within 3 s) by appropriate tilt (30°), and there was virtually no fluid imprinting on SSC-COOH surfaces (Fig. S5). In contrast, all of the tested droplets spread on the unmodified PG surface and left numerous traces. This once again proved that the SSC-COOH surface exhibits exceptional “slippery” performance for its application to various aqueous-based liquids. In addition, the application of SSC to other medically related metal and polymer surfaces such as copper, aluminum, silicon (Si), polyisoprene (rubber), polyethylene terephthalate (PET), etc. (Fig. S6), also showed exceptional “slippery” performance. This not only showed the potential for SSC broad-spectrum applications, but also further confirmed that the “slippery” property of the SSC modified surfaces are caused by the coating itself, independent of the substrate. Combined with the above results, consistent with LIS and SLIPS modified surface, SSC-COOH did not have very large CA for all liquids, but the SA and CAH were very small, and showed exceptional dynamic slippery performance.

In order to further verify that the anti-adhesion properties of the modified surface may benefit from the reduction of the substrate adhesion (i. e., adhesion work) on the SSC-COOH modified surface, the surface free energy is calculated according to the Owens-Wendt-Rabel-Kaelble (OWRK) method (ISO 19403-2-2017). Combining the Young equation and the Van Oss equation, the work of adhesion can be performed as:

$W_{a\;}={2\left(\gamma_{l}^{p}\gamma_{s}^{p}\right)}^{\frac{1}{2}}+{2\left(\gamma_{l}^{d}\gamma_{s}^{d}\right)}^{\frac{1}{2}}=\gamma_{l}\left(1+\cos\;\theta \right)$ [38]

Among them, $W_{a}$ is the work of adhesion, *θ* is the contact angle for the liquids. $\gamma_{l}$, $\gamma_{l}^{p}$ and $\gamma_{l}^{d}$ are the surface tension of the respective liquid and the polar and dispersive fractions of the surface tension, respectively. $\gamma_{s}^{p}$ and$\gamma_{s}^{d}$ are the polar and dispersive fractions of the surface tension of the solid surface, respectively. Since the corresponding γ_l_ of each liquid is a measured value (e. g., γ_l_ = ∼72.8 mJ/m for DI water, γ_l_ = ∼40 mJ/m for bacterial solution, γ_l_ = ∼52.1 mJ/m for DEME +10 % FBS and γ_l_ = ∼60 mJ/m for blood), The theoretical calculation results were shown in Fig. 2i. The results showed that SSCMC can significantly reduce the surface adhesion energy, thus showing exceptional “slippery” characteristics, which will provide an important basis for effectively preventing the adhesion of biofouling.

### 2.2 The mechanism and performance of stability of SSCMs

Subsequently, the stabilization mechanism and performance of the nanohesive-based SSC-COOH was explored. All samples were dealt with blood flow shock challenge (high physiological shear rate ∼ 1750 s^−1^, simulated blood, a viscosity of approximately 4 mPa s) [39]. Firstly, to verify whether the robust stability of SSC-COOH is caused by the formation of molecular forces between the amino-functionalized SiO_2_ nanoparticles and the silicone oil. We conducted two sets of experiments with -OH and -OCH_3_ instead of -COOH terminated silicone oil, and made different types of solid coating (SSC-OH, SSC-OCH_3_): 1) Try to destroy the electrostatic interactions by erosion of the ionic solution and observe their stability properties. SSC-COOH was immersed in NaCl, CaCl_2_ and FeCl_3_ solutions (all at a concentration of 1 M) under 1750 s^−1^ shear rate for 6 h and 24 h, respectively, while SSC-OH and SSC-OCH_3_ were used as control groups. 2) Explore the effects of hydrogen bonds formation. SSC-COOH and SSC-OH was suffered neutral high-speed blood flow shocks (1750 s^−1^, for 3 days and 7 days), with SSC-OCH_3_ as the control group. All experiments evaluated the stability of different coatings with changes in DI water CA, SA and CAH.

**Fig. 3:**
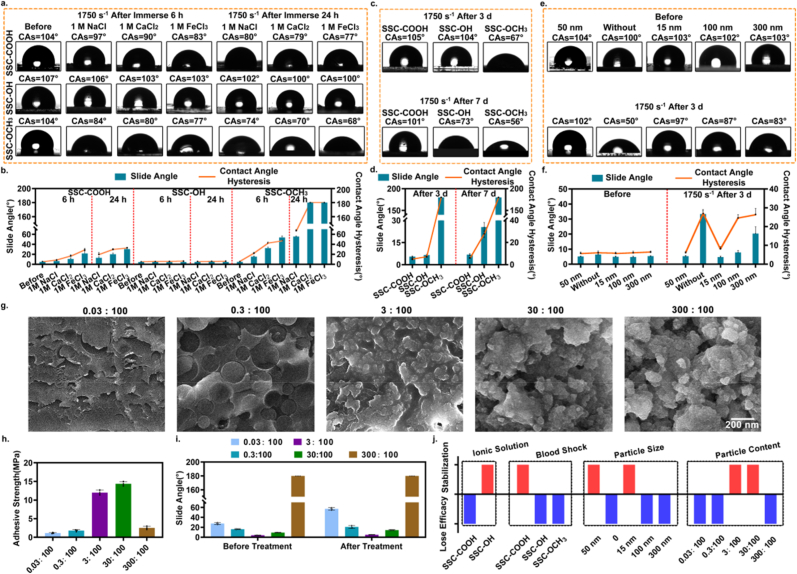
**The mechanism of stability and long-term durability of SSCMs.** a) Wetting phenomena variation of DI water (2 μl) on the SSC-COOH, SSC-OH and SSC-OCH_3_ surfaces that after immerse in the different ionic solutions (NaCl, CaCl_2_ and FeCl_3_) at 1 M concentration under 1750 s^−1^ shear rate for 6 h and 24 h, respectively. b) Statistics of the SA and CAH of the DI Water (10 μl) on the surfaces of SSC-COOH, SSC-OH and SSC-OCH_3_. c) Wetting phenomena of DI Water (2 μl) on the three surfaces (including SSC-COOH, SSC-OH and SSC-OCH_3_) after treatment for 3 days and 7 days at 1750 s^−1^ blood impact, respectively. d) Statistics of the SA and CAH of the above three surfaces for DI Water (10 μl). e) Wetting phenomena variation of DI water (2 μl) on the different size of SiO_2_ particles and without SiO_2_ particles of SSC-COOH surfaces before and after treatment for 3 days at 1750 s^−1^ blood flow impact, respectively. f) Statistics of the SA and CAH of the DI Water (10 μl) on the above difference surfaces, where 180° indicates that the droplets in the corresponding surfaces showed diffusion and wetting phenomena, cannot be measured the specific value. After adjusting the ratio of nanoparticles to silicone oil, g) the cross-sectional structure diagrams of each group of nanoparticles, as well as the changes in h) adhesion strength and i) sliding performance. j) Diagram summarizing the stabilization or lose efficacy results of different surfaces after different treatments impact (including ionic solutions, blood shock, particle content and particle size). Error bar represents the mean ± SD. N = 4, averaged.

As shown in Fig. 3a–d, SSC-OCH_3_ exhibited significant declines in hydrophobic and sliding properties after the above-mentioned fluid impacts. The results might be attributed to the fact that there was only a weak van der Waals forces between silicone oil and nanoparticles, resulting in the coating being prone to failure in fluid environments. Compared with SSC-OH, the hydrophobic and slippery properties of SSC-COOH decreased significantly with the increase of ionic strength and time. The results showed that due to the presence of ions, the electrostatic interaction between silicone oil and nanoparticles in SSC-COOH was disrupted, and SSC-COOH gradually lost its stability in ionic solutions [32,40]. However, after the long-term neutral blood flow challenge (7 days), the hydrophobic and sliding properties of SSC-COOH remained basically unchanged. On the contrary, the performance of SSC-OH decreased significantly. It is worth noting that when the impact time was reached 3 days, the hydrophobic and sliding properties of SSC-OCH_3_ decline significantly, while SSC-OH remains basically unchanged. This may be attributed to the formation of hydrogen bonds, which is conducive to the stability of the coating (Fig. S7). The above results indicated that both electrostatic interactions and hydrogen bonds have significant impacts on the stability of the coating (Fig. S8). However, the stability of SSC-COOH in neutral fluid solutions is much greater than that of SSC-OH. The stability experiments of the coatings indicated that the robust stability of SSC-COOH is mainly based on electrostatic interactions, with hydrogen bonds having a secondary influence on stability. In addition, the stability effects of electrostatic interactions and hydrogen bonds on SSC were further analyzed from the perspective of thermal stability. As shown in Fig. S9, thermogravimetric analysis results showed that the initial decomposition temperature of the SSC-COOH system increased to 360 °C compared with that of -COOH terminated silicone oil (232 °C). In the SSC-OH system, the initial decomposition temperature of SSC-OH (380 °C) was slightly higher than that of -OH terminated silicone oil (320 °C). However, the initial decomposition temperature of SSC-OCH_3_ and -OCH_3_ terminated silicone oil was between 350 and 370 °C. The results showed that SSC-COOH exhibits significantly increased thermal stability, which may be due to the strong electrostatic interactions between the amino-SiO_2_ and the -COOH terminated silicone oil. Similarly, for the SSC-OH system, the thermal stability was slightly improved, which may be attributed to the formation of hydrogen bonds. While in SSC-OCH_3_, there was no chemical bonds between the nanoparticles and silicone oil, and its thermal stability was not significantly different from that of -OCH_3_ terminated silicone oil [41].

Furthermore, the effect of the nanoparticles on the coating stability was further explored. Using -COOH terminated silicone oil as lubricant, different SSC-COOH were prepared by regulating the size of functional nanoparticles (15, 100, 300 nm), with high-speed fluid impact challenge (1750 s^−1^ shear rate for 3 days). Similarly, the stability of the coating was evaluated against the changes in water CA, SA and CAH properties, and SSC without nanoparticles were used as a control group. As shown in Fig. 3e and f, 50 nm-SSC-COOH and 15 nm-SSC-COOH can still maintain their original hydrophobic and slippery properties after blood impact. However, without nanoparticles, 100 nm-SSC-COOH and 300 nm-SSC-COOH showed different degrees of loss of hydrophobic and slippery properties. It can be seen that, compared with no nanoparticles, 15 nm-SSC-COOH and 50 nm-SSC-COOH exhibit exceptional stability, which may be due to the presence of nanoparticles that cross-link to each other to form a dense microstructure, thus improving the mechanical stability (resistance to fluid shock) [33]. Furthermore, with increasing nanoparticle size, the stability is seriously affected, which may be caused by the adhesion energy of the coating and the interface is reduced [31].

In addition, the influence of nanoparticle concentration on the SSC-COOH coating performance was further discussed. Different SSC-COOH were prepared by adjusting the mass ratio of nanoparticles to silicone oil. Before discussing the stability performance, the structural changes and “slippery” performance of SSC-COOH were studied, as this characteristic is an important prerequisite for achieving anti-biological adhesion. Fig. 3g presented the SEM images of the internal cross-sections of SSC-COOH with three mass ratios. Compared with the dense structure formed by the initial mass ratio (3:100) of SSC-COOH, the low proportion (0.03:100, 0.3:100) group showed structural delamination/cracking, while the high proportion (30:100, 300:100) SSC-COOH presented a loose structure. These two structural types may be detrimental to the basic performance and stability of SSC-COOH. To further verify the above conjecture, we conducted “slippery” performance tests on three groups of SSC-COOH. The results (Fig. 3h and i) showed that compared with the initial mass ratio (3:100) SSC-COOH group, the SA (DI water) of the low proportion group decreased significantly to ∼28°. This might be due to the excessively low content of aminated nanoparticles, the limited effective capture of silicone oil through electrostatic interactions, and the affected sliding performance. However, in the test, the high proportion group lost the original “slippery” advantage due to the loose distribution of nanoparticles. These results indicate that the appropriate proportion of nanoparticles significantly affects the structural stability and “slippery” advantage of SSC-COOH (Fig. 3j). Therefore, considering factors such as stability and “slippery” performance, in this work, the ∼50 nm nanoparticles and a mass ratio of nanoparticles to silicone oil of 3:100 were selected.

### 2.3 Anti-adhesion properties of SSC-COOH modified catheter (SSCMC)

Under the influence of various components in the human body, such as proteins, bacteria, cells, platelets, etc., the implantable devices, especially the blood-contacting biomedical catheters, are very susceptible to biofouling, such as protein adhesion, which can trigger the recruitment of immune cells [42]. Which not only narrows the safe retrieval window of the device, but even triggers a nonspecific host cell immune response in the body, resulting in tissue fibrosis [43]. To evaluate the biofouling resistance of the SSCMC, the adhesion behavior of proteins, bacteria and cells on the modified surfaces were investigated separately.

**Fig. 4:**
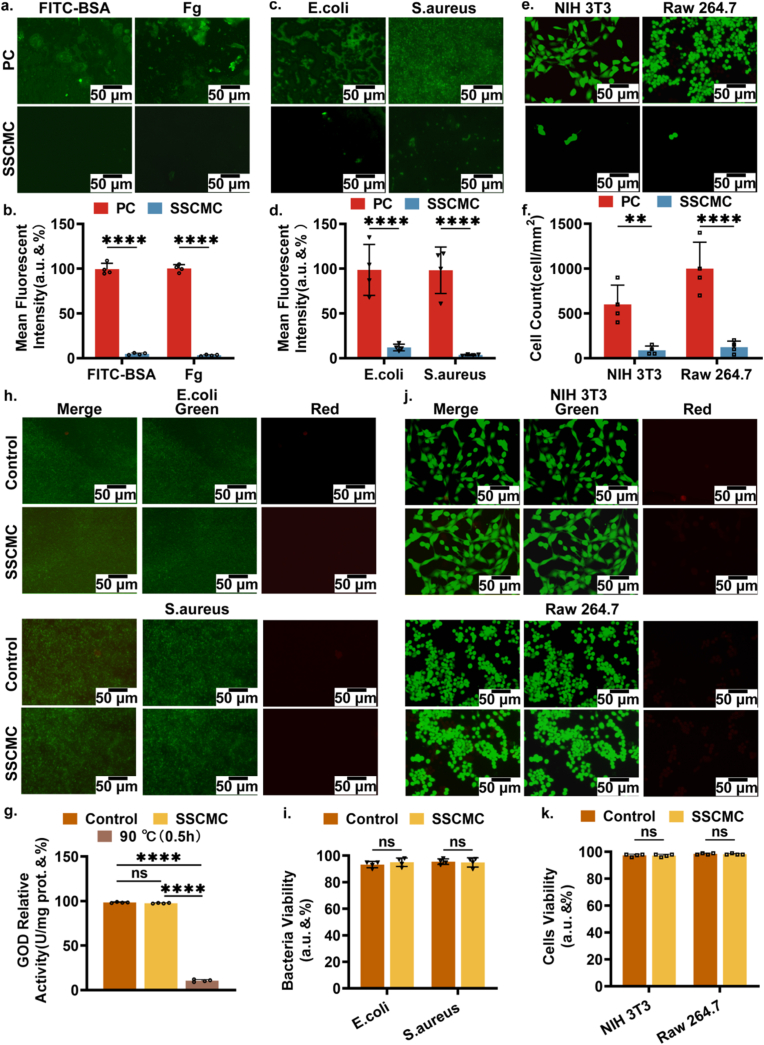
**Anti-adhesion Properties of SSCMC.** a) Protein molecule adhesion fluorescence plots of FITC-BSA and Fg on two surfaces. b) Quantitative analysis of the amount of adhesion of FITC-BSA and Fg on the two surfaces showed significant differences. c) Bacterial adhesion fluorograms of *E. coli* and *S. aureus* on two surfaces. d) Results of quantitative analysis of the amount of adhesion of *E. coli* and *S. aureus* on both surfaces showed significant differences. e) Cell adhesion fluorograms of NIH 3T3 and Raw 264.7 on both surfaces. f) Results of quantitative analysis of the amount of adhesion of NIH 3T3 and Raw 264.7 on both surfaces, with significant differences. Fluorograms and quantitative analysis results exploring the effect of SSC on the activity of two bacterial species. g) Results of quantitative analysis examining the effect of SSC surfaces on the activity of protein. h) Fluorograms examining the effect of SSC on the activity of two bacteria. i) Results of quantitative analysis examining the effect of SSC on the activity of two bacterial species. j) Fluorograms examining the effect of SSC on the activity of two cell types. k) Results of quantitative analysis exploring the effect of SSC on the activity of NIH 3T3 and Raw 264.7. Error bar represents the mean ± SD. Significance was calculated by one-way analysis of variance. ∗∗*p* < 0.01, ∗∗∗∗*p* < 0.0001, ns: not significant. N = 4, averaged.

First, fluorescein isothiocyanate bovine serum albumin (FITC-BSA, the protein concentration was about 5.12 mg/ml) and fluorescent fibrinogen (Fg, the protein concentration was about 5 mg/ml) were used as representative proteins to investigate the resistance of SSCMC to proteins. At the same time, unmodified PC samples were used as control groups. Fig. 4a showed representative images of protein molecules adhering to a variety of sample surfaces after 24 h incubation. Based on the fluorescence images, most areas of the PC surface showed bright green fluorescence (FITC-BSA and Fg), indicating that proteins were more likely to adhere to the surface of catheters. On the contrary, the SSCMC surface was almost black with no fluorescence at all, and further statistics showed that compared with that on the PC surface, the adhesion of FITC-BSA and Fg on the SSCMC surface were reduced by 94.9 % and 97.1 %, respectively (Fig. 4b), suggesting that the “solid-like” slippery coating on the surface of catheters significantly inhibits the adhesion of proteins. As a key step in the inhibition of biofouling and foreign body reactions, SSC-COOH efficiently inhibits protein adsorption, which may be a new and ideal strategy for implantable devices to eliminate biofouling, bacterial and cellular adhesion.

Second, we continued to investigate whether the modification method could prevent bacterial adhesion, thereby inhibiting bacterial biofilm formation at the source and reducing bacterial contamination on the surface of clinical medical devices and the risk of infection for patients. SSCMC was co-incubated with two clinically important biofilm-forming pathogens, *Escherichia coli* (*E. coli*) and *Staphylococcus aureus* (*S. aureus*), in a static incubation at 37 °C, while unmodified PC was used as control group. Fig. 4c showed the representative images of bacterial adhesion on different sample surfaces. After 24 h of incubation, the surface of PC was covered by a large number of bacteria. In stark contrast, the SSC modified surface showed only low-intensity green fluorescence, indicating its strong resistance to bacterial adhesion. Further statistics showed that the normalized fluorescence intensity of the SSCMC against bacterial adhesion was reduced by at least 86.7 % (*E. coli*) compared to the PC group (Fig. 4d).

Implantable medical catheters and other blood-contacting biomaterials can be recognized by blood cells, thus stimulating immune cells such as macrophages to recognize, adhere to, and accumulate on the surface of the implanted device, inducing an inflammatory response and promoting thrombosis [44]. Therefore, we further explored the resistance of SSCMC to cell adhesion. Embryonic fibroblast NIH 3T3 and monocyte-macrophage Raw 264.7 were used as representative cells to assess resistance to cell adhesion *in vitro*. Meanwhile, unmodified PC was used as the control group. After 24 h of incubation at 37 °C, cells were labeled with the green fluorescent dye Calcein acetoxymethyl ester (CAM) and visually observed under a fluorescence microscope. As shown in Fig. 4e and f, similar to bacterial adhesion, both NIH 3T3 and Raw 264.7 covered the unmodified PC surface in large number, whereas only sporadic green fluorescent cell adhesion was observed on the SSCMC surface. Counting the number of fluorescent cells anchored to the catheter surface per unit area, the average cell number in the SSCMC group (87.5 cells/mm^2^ for NIH 3T3 and 122.5 cells/mm^2^ for Raw 264.7) was at least 5.8 times lower than that in the PC group (600 cells/mm^2^ for NIH 3T3 and 1000 cells/mm^2^ for Raw 264.7). Which indicated that the SSC surface has outstanding anti-cell adhesion properties.

To test whether the resistance of SSCMC to biofouling is due to its toxicity, several further activity assays were carried out. Firstly, the effect of SSCMC on protein activity was evaluated by using a representative protein, glucose oxidase (GOD). The SSCMC was co-incubated with GOD at 37 °C for 24 h. After the incubation was completed, the GOD activity was detected by the GOD activity assay kit. Meanwhile, blank treatment and heat denaturation treatment were used as control groups. As shown in Fig. 4g, SSCMC had no significant effect on protein activity compared to the blank control group, while heating almost eliminated protein activity. Subsequently, SSCMC was co-incubated with *E. coli* or *S. aureus* in porous plates at 37 °C for 24 h, and the medium without SSCMC served as the control group. The results showed that red fluorescence associated with dead bacteria was hardly observed in all groups (Fig. 4h and i), indicating that SSCMC did not affect bacterial viability. Statistical results showed that the bacterial activity of the SSCMC group was more than 94 %, which was not significantly different from the control group. Similarly, SSCMC were co-incubated with NIH 3T3 or Raw 264.7 in porous plates for 24 h at 37 °C, and those without SSCMC served as the control group. The effect of SSCMC on cell activity was assessed by determining cell viability at the bottom of the plate using a live/dead staining kit. Representative fluorescence images were shown in Fig. 4j, and similar to the bacterial activity results, no obvious red fluorescent cells were seen in all media, regardless of the presence or absence of SSCMC. Statistical results showed that cell viability in the presence of SSCMC was greater than 97 % for both cell types (Fig. 4k), indicating that SSCMC do not affect cell viability. These results indicated that the outstanding anti-biofouling properties of SSCMC do not depend on protein inactivation or killing bacteria or cells to cause them. Which means that SSC can serve as a biocompatible interface in medical practice, which plays a critical role in the design of anti-biofouling implantable devices for clinical translation.

As mentioned above, any aqueous-based liquid including DEME containing proteins or even extremely complex blood can easily slide off from the SSCMC surface without any residue, and this exceptional slippery property makes it difficult for proteins to interact with the SSCMC coating in any force. Thus, protein molecules are easily cut off by the dynamic flow of the liquid and even diffuse into the medium under static conditions. Furthermore, the adhesion of bacteria and cells on the surface of the substrate is often mediated by stick to the bacterial surface or the protein-ligand integrin embedded in the cell membrane, which is mainly composed of proteins, or and polysaccharide molecules [45]. Benefiting from the exceptional “slippery” property of SSCMC, bacteria or cells are prevented from attaching to smooth surfaces by cilium, pseudopods or cell membrane proteins and other mechanisms [46], which effectively inhibits the adhesion of bacteria and cells to SSCMC surface.

### 2.4 Test of antithrombosis properties of SSCMC *in vitro* and *in vivo*

**Fig. 5:**
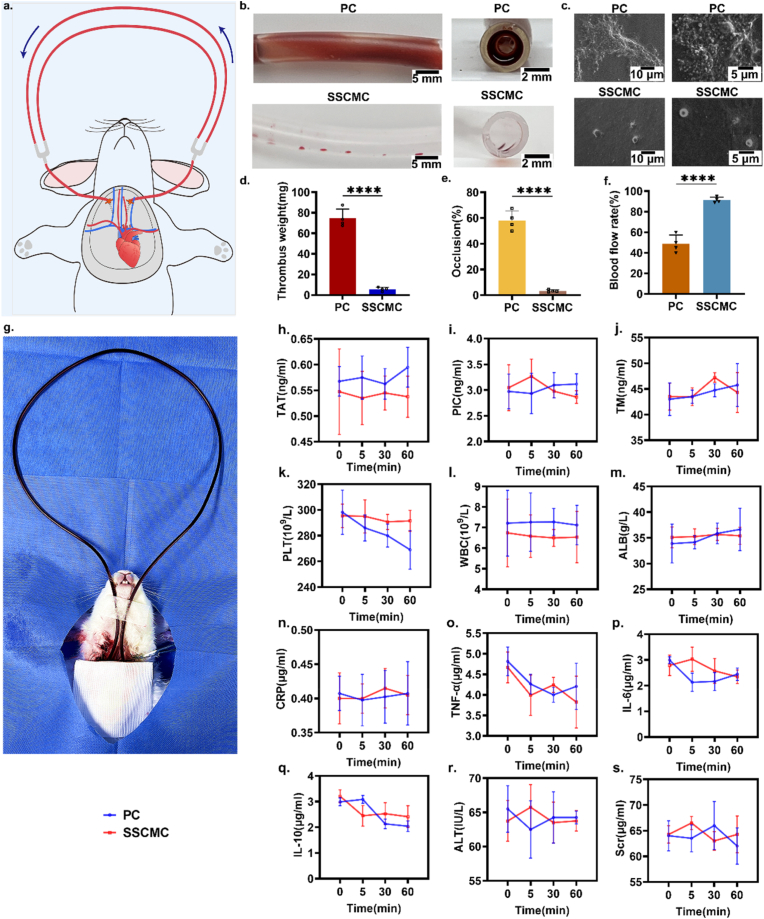
**Test of antithrombosis properties of SSCMC *in vitro* and *in vivo*.** a) Surgical schematic of *in vitro* blood circulation in rabbits. b) Diagrams of blood cells adhering to the inner lumen of both catheters before and after modification of *in vitro* blood circulation for 2 h. c) Comparison of blood cells adhering to the surfaces of both catheters before and after modification of the inner lumen of both catheters under SEM. d) ∼ f) Quantification of thrombus weight, lumen occlusion and blood flow rate formed in the lumen of the two catheters, respectively. g) Blood circulation modeling to perform blood correlation analysis. h) Thrombin-antithrombin III enzyme complex (TAT) (an early marker of coagulation activation). i) Tissue-type fibrinogen activator-inhibitor 1 complex (PIC) (risk indicator for venous thromboembolism). j) Thrombomodulin (TM). k) Platelets (PLT). l) White blood cells (WBC). m) Serum albumin (ALB). n) Acute inflammation indicator c-reactive protein (CRP). o) Tumor necrosis factor (TNF-α). p) IL-6. q) IL-10. r) Liver function indicator alanine aminotransferase (ALT). s) Kidney function indicator serum creatinine (Scr). Error bar represents the mean ± SD. Significance was calculated by one-way analysis of variance. ∗∗∗∗*p* < 0.0001. N = 4, averaged.

Thrombosis prevention is a key indicator for the clinical use of blood-contacting biomaterials and other implanted medical devices. Modification of the surface properties of the device, such as inhibition of fibrin and platelet adhesion, is expected to inhibit clot formation [47]. Given the strong ability of SSCMC surfaces to prevent biological contamination, it was investigated whether the SSCMC could be resistant to thrombosis. To be more clinically relevant, the inhibition of blood contamination and thrombosis by the SSCMC was investigated by *in vitro* blood circulation experiments in catheters (Fig. 5). PVC catheters before and after SSC treatment were assembled with commercially available medical tubing and connected to rabbit arteriovenous shunt circuits (Fig. 5a). After 2 h of *in vitro* blood circulation without the use of anticoagulants, all catheters were collected to observe thrombus formation in the catheter circuits and to determine surface blood cell adhesion, thrombus weight, catheter occlusion rate, and blood flow velocity to evaluate their antithrombotic properties. As shown in Fig. 5b, in the unmodified PC group, there was a large amount of coagulation, whereas SSCMC was almost transparent and no coagulation formed, indicating highly efficient thrombosis inhibition. Surface adhesion analysis by SEM further confirmed that the presence of SSC effectively prevented thrombus formation. As shown in Fig. 5c, a large number of erythrocytes and platelets, and even fibrous thrombus, aggregated on the unmodified PC group, whereas the blood contact surface of the SSCMC group showed only sporadic adhesion of blood cells. In the analysis of the weight of the thrombus, the total weight of the thrombus in the SSCMC circuit was at least 12 times less than in the unmodified PC group (Fig. 5d). Using image analysis techniques to quantify the degree of channel occlusion after circulation of the two catheters, the occlusion of the unmodified catheters was nearly 60 % after only 2 h of blood circulation, whereas the occlusion of the SSCMC was only 3.3 % (Fig. 5e). Under the same pressure pumping conditions, the blood flow rate of the SSCMC improved significantly and remained as high as 91 % compared to the pre-circulation period, while the bare tube had dropped to approximately 48 % (Fig. 5f). These results suggested the SSC could prevent proteins and cells adhesion not only *in vitro* but also *in vivo* and hence reduce thrombosis formation, which is a challenging task for most existing blood-contact materials and implantable devices.

For implantable biomedical devices such as central venous catheters, in addition to preventing thrombosis, the safety for *in vivo* use during prolonged or extensive blood exposure must be considered, which requires a comprehensive assessment of their effects on blood composition and liver/renal function in a systematic manner. Blood compatibility is one of the important indicators.

Blood compatibility of SSC was evaluated by *in vitro* hemolysis test using New Zealand rabbit blood. The experimental results showed (Fig. S10) that after incubation with red blood cell suspension for 1 h in each group, the hemolysis rate of the MC group was 3.15 % (<5 % indicated good blood compatibility) [48], which also confirmed that SSC-COOH surface has good blood compatibility and fully meets the requirements of clinical application.

Next, SSCMC was selected to simulate clinical use and was placed in the rabbit arteriovenous shunt circuit with an unmodified PC as a control (Fig. 5g). At predetermined time intervals (after 0, 5, 30, and 60 min of circulation), rabbit blood was collected for biochemical analysis, including thrombin-antithrombin complex (TAT, CUSABIO), fibrinolytic-α2 fibrinolytic inhibitor complex (PIC, mlbio), thrombomodulin (TM, mlbio), platelet (PLT), white blood cell (WBC), serum albumin (ALB, proteintech), C-reactive protein (CRP, proteintech), tumor necrosis factor-α (TNF-α, proteintech), and inflammatory and immunosuppressive factors (IL-6, IL-10, proteintech) to assess physiological parameters such as coagulation, inflammatory response, and organ function. After cycling, the relevant coagulation characteristic parameters (including TAT, PIC, and TM), were varied within the normal range of the SSCMC group. In contrast, the parameters of PC group showed an increasing trend, indicating a tendency for higher coagulation after prolonged exposure to blood (Fig. 5h–j). Moreover, the PLT levels gradually decreased in the PC group, whereas they remained essentially unchanged in the SSCMC group. This difference may be attributed to the occurrence of coagulation, where a large number of platelets in the PC group adhered to the surfaces of the catheter, thereby reducing the number of platelets in the circulating blood (Fig. 5k). In addition, assessment of the inflammatory response showed no significant changes in pro-inflammatory parameters, including WBC, CRP, TNF-α, IL-6, and IL-10, in either PC or SSCMC. As shown in Fig. 5l–q, these results indicated that there was no pro-inflammatory tendency of the catheters in either group. In addition, the pro-inflammatory indices in the SSCMC group were consistent with the results of the clinically used PC group, suggesting that the SSC surface did not further enhance the inflammatory response of the material. The potential toxicity of the materials and coatings to organs and tissues was further evaluated by measuring the blood concentrations of the liver enzyme alanine aminotransferase (ALT, CUSABIO) and the kidney parameter serum creatinine (Scr, Beyotime). As shown in Fig. 5r and s, there was no significant change about ALT and Scr in all groups, indicating that PC and SSCMC were not toxic to tissues and organs during the circulation period. There was also no significant difference in SSCMC compared to PC, further confirming the biosafety of SSC.

Then, FITC-Fg was added into fresh rabbit blood and cardiopulmonary bypass was performed for 1 h to observe the adhesion of fibrin network on SSC surface. As shown in Fig. S11, compared with the closed-loop system composed of PC, there was less fluorescent fibrin on the surface of SSC, which meant that the exceptional “slippery” performance of SSC could effectively resist the adhesion of fibrin. In addition, the anticoagulation function of SSC was studied by *in vitro* coagulation time. The results showed that (Fig. S12) the blood in the unmodified PC was completely coagulated at 35 s, while the coagulation time of the centrifuge tube modified by SSC could be extended to 2623 s, indicating that SSC has potential application prospects in anticoagulation.

### 2.5 The biosafety of SSCMC *in vivo*

**Fig. 6:**
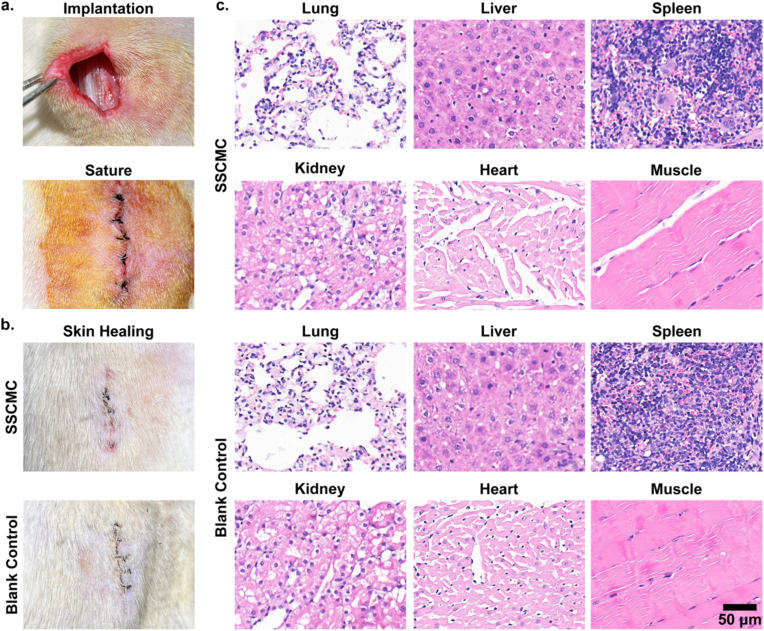
**The biosafety of SSCMC *in vivo*.** a) Procedures of subcutaneous implantation on the back of rats. b) Skin healing of rats in both groups 7 days after subcutaneous implantation. c) H&E staining results of lung, liver, spleen, kidney, heart and muscle tissue at subcutaneous implantation site.

To further evaluate the tissue safety performance of SSC *in vivo*, we implanted SSCMC into the subcutaneous tissue of SD rats for 7 days to observe skin healing, acute inflammatory responses, and early foreign body reactions. Meanwhile, an antibacterial original catheter was used as a control group. As shown in Fig. 6, regardless of whether the coating was applied or not, complete healing of the surgical site skin was observed in all rats after 7 days, with no signs of abscesses, inflammatory exudation, or other adverse reactions around the implantation area. In addition, the hematoxylin-eosin (H&E) staining results showed that after subcutaneous implantation of the material, no obvious infiltration of inflammatory cells (such as neutrophils, lymphocytes or macrophages) was observed in various tissues including the lung, liver, spleen, kidney, heart and muscle. The tissue morphology and structure were intact, with cells arranged in an orderly manner. No pathological changes such as edema, necrosis or fibrosis were observed, suggesting that both the cell morphology and quantity were in a normal physiological state. The above results indicate that SSC does not cause obvious inflammatory or foreign body reactions *in vivo*, further confirming its good biocompatibility.

### 2.6 Test of the durability of SSCMC

In clinical practice, most blood-contacting devices need to remain in the body or even blood vessels for a long period of time. Therefore, the stability of the coating plays a crucial role in the long-term efficacy of the device. Here, the stability of the prepared SSCMC in dynamic blood flow and its long-term resistance to biofouling were studied.

**Fig. 7:**
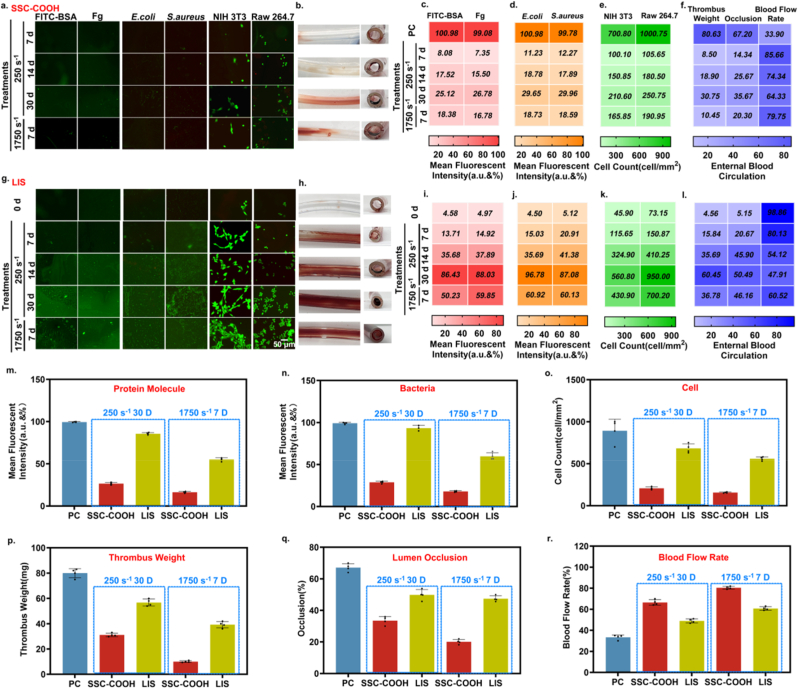
**Test of durability of SSCMC.** a) Representative fluorescence images of SSC-COOH modified catheter samples incubated with proteins, bacteria, and cells for 3 days after blood flow low shear rate environment treatment for 7, 14, 30 days, and high shear rate environment impact for 7 days, respectively. b) After 2 h of *in vitro* blood circulation, the thrombus formed in the inner lumen of the catheters. c) ∼ e) The adhesion results of proteins, bacteria and cells of the unmodified PC group and the SSCMC group after the various treatments mentioned above were quantitatively analyzed, and there were significant differences. f) The weight of the thrombus formed in the inner lumen of the catheters, occlusion, and blood flow rate of each SSCMC were quantified, and significant differences were found. g) Representative fluorescence images of LIS modified catheter samples incubated with proteins, bacteria, and cells for 3 days after blood flow low shear rate environment treatment for 7, 14, 30 days and high shear rate environment impact for 7 days, respectively. h) After 2 h of *in vitro* blood circulation, the thrombus formed in the inner lumen of the catheters. i) ∼ k) The adhesion results of proteins, bacteria and cells of LIS groups after the various treatments mentioned above were quantitatively analyzed. l) The weight of the thrombus formed in the inner lumen of the catheters, occlusion, and blood flow rate of each group were quantified. m) ∼ o) The adhesion results of proteins, bacteria and cells of the unmodified PC, SSC-COOH and LIS groups after the blood flow low shear rate environment treatment for 30 days, and high shear rate environment impact for 7 days were quantitatively analyzed. p) ∼ r) The weight of the thrombus formed in the inner lumen of the catheters, occlusion, and blood flow rate of each group after above treatments were quantified. Error bar represents the mean ± SD. Significance was calculated by one-way analysis of variance. ∗∗∗∗*p* < 0.0001. N = 4, averaged.

To simulate the blood flow environment, SSCMC samples were placed in a microfluidic device for blood flow shock challenge (simulated blood, a viscosity of approximately 4 mPa s). The long-term anti-protein, bacterial and cell adhesion, and anti-thrombotic properties of SSCMC were investigated at physiological shear rate [39] (∼250 s^−1^, for 7, 14, and 30 days & ∼ 1750 s^−1^, for 7 days). Meanwhile, the lubricating surface LIS modified catheter suffered the same blood flow environment challenge as a control group. All SSCMC, LIS and unmodified PC samples after fluid challenge were incubated with proteins (FITC-BSA and Fg), bacteria (*E. coli* and *S. aureus*), cells (NIH 3T3 and RAW 264.7) for 3 days, and the adhesion level on the catheter surface was recorded using fluorescent labeling. As shown in Fig. 7a, under prolonged blood environmental challenges (∼250 s^−1^, 30 days & ∼ 1750 s^−1^, 7 days), SSCMC showed only a weak green fluorescence and still maintain the advantage of resistance to biofouling when compared to PC (Fig. S13). Statistical results showed that (Figures S14, 7c ∼ 7e and 7m ∼ 7o), the adhesion to proteins, bacteria and cells was still reduced by at least 71 % compared with PC. In stark contrast, As the blood flow challenge time or shear rate increases, the surface of the LIS coating modified catheter showed large amounts of green fluorescent protein, bacteria and cells (Fig. 7g), which indicated that the LIS modified catheter has gradually failed (<28 %), which may be caused by the gradual loss of LIS surface lubrication under blood flow challenge (Figures S15, 7i ∼ 7k).

Similarly, in the antithrombotic experiment, SSCMC and LIS treated with different conditions were extracorporeal for 2 h, the thrombus formation in the catheter was observed and the thrombus weight, occlusion rate and blood flow rate were measured. As shown in Fig. 7b, f and 7p ∼ 7r, the antithrombotic efficiency of SSCMC decreased slightly with increasing fluid challenge time, and remained fairly effective (>67 %) as assessed by thrombus weight, occlusion rate and blood flow rate (∼250 s^−1^, for 30 days & ∼ 1750 s^−1^, for 7 days). The cross section of SSC antithrombotic experiment observed by SEM showed that the coating was not significantly damaged (Fig. S16), which provided the possibility of realizing long-term inhibition of thrombosis and biological contamination in fluid environment. Moreover, LIS modified catheters showed significant clotting after blood flow challenges (Fig. 7h and l).

Furthermore, the effect of various physiological conditions on the stability of the coating was explored. The SSCMC was placed in a blood flow environment of pH = 1, pH = 5.5, pH = 7.5, 0.9 % NaCl (250 s^−1^, 30 days), and characterized by slippery performance (SA, CAH) and resistance bioadhesion (proteins) performance. As shown in Fig. S17, the slippery properties and anti-adhesion properties of the SSCMC surfaces did not change significantly under nearly neutral physiological conditions (pH = 5.5, pH = 7.5, 0.9 % NaCl). In contrast, in the strongly acidic environment, its slippery properties and anti-adhesion properties decreased sharply, which may be that the strongly acidic environment breaks the electrostatic interactions of the coating [49]. The above results indicated that SSC-COOH is not suitable for long-term use in extremely acidic environments, but the advantage of long-term stability against biological adhesion can still be achieved under most of the physiological conditions.

In addition, the wear stability of the coating is particularly important. The changes in the smoothness of SSC-COOH were observed respectively by simulating the normal frictional force of the vascular wall with sandpaper (the frictional force was approximately 10 Pa), cross-cutting operation, high-pressure treatment for 60 min, and ultrasonic treatment for 30 min. The stability and wear resistance of SSC-COOH were studied. The results (Fig. S18) showed that after the wear treatment under the above different conditions, the SA of SSC-COOH was still less than 10°, maintaining excellent sliding performance. This indicated that SSC-COOH can effectively resist the wear treatment in various normal clinical application environments and has excellent long-term stability and wear resistance.

These results demonstrated that SSCMC has exceptional, ultra-stable, and robust *anti*-bioadhesion, antithrombotic and mechanical stability properties in dynamic fluid environments. The outstanding stability characteristics and improved technology have a wide range of potential applications not only for antithrombotic vascular catheter, but also for maintaining long-term performance in fluid environment scenarios such as lossless liquid transport.

## 3 Conclusion

In summary, we have developed a promising strategy for comprehensive nanohesive-based “solid-like” slippery coating acting on blood-contacting biomedical devices (such as medical catheters) to reduce biofouling, inhibit thrombosis and infection. The coating is formed by functionalized nanoparticles capturing lubricating oil and embedded inside and surface of epoxy resin. After coating modification, benefiting from the slippery properties of SSC, SSCMC can effectively prevent aqueous-based liquids, proteins, bacteria, cells and platelets adhesion. It also can inhibit thrombosis in rabbit blood circulation and does not cause inflammation, and implantable experiments showed that it does not cause damage to surrounding tissues, with outstanding biocompatibility. The anti-adhesive and antithrombotic properties of SSCMC still exceed 71 % and 67 % under long term low shear rate (250 s^−1^, for 30 days) or high shear rate (1750 s^−1^, for 7 days) blood flow environment, with exceptional durability and firmness. Stability mechanism exploration experiments confirmed that functionalized nanoparticles are the basis of coating stability. Through electrostatic interactions and other forces, in conjunction with epoxy resin encapsulation, -COOH terminated silicone oil is strongly trapped. In addition, the dense microstructure formed by the cross-linking of nanoparticles significantly improves the mechanical strength of the coating. The synergistic effect of these two mechanisms enables SSC to maintain its advantages of robustness and ultra-stability in the blood flow environment. We anticipate that the nanohesive-based “solid-like” slippery coating will not only be a promising candidate for designing blood-contacting biomedical devices to prevent thrombosis and biofouling, but also open new horizons for the development of medical implants in various static or fluid environments of persistent and exceptional biofouling-resistance.

## CRediT authorship contribution statement

**Shu Zhang:** Writing – original draft, Visualization, Validation, Software, Methodology, Investigation, Formal analysis, Data curation. **Jihua Zou:** Visualization, Software, Resources, Methodology, Investigation, Data curation. **Yupeng Xiao:** Visualization, Software, Methodology, Formal analysis, Data curation. **Xiaoying Qiu:** Visualization, Software, Methodology. **Yao Shen:** Software, Project administration, Methodology. **Yijin Zhao:** Software, Methodology, Formal analysis. **Tao Fan:** Software, Project administration, Methodology. **Manxu Zheng:** Software, Methodology, Formal analysis. **Guozhi Huang:** Visualization, Validation, Investigation, Funding acquisition, Conceptualization. **Qing Zeng:** Writing – review & editing, Visualization, Validation, Supervision, Investigation, Conceptualization. **Chengduan Yang:** Writing – review & editing, Visualization, Validation, Supervision, Resources, Investigation, Funding acquisition, Data curation, Conceptualization.

## Statistical analysis

Each experiment was performed in at least four replicates. Data are presented as the mean ± standard deviation (mean ± SD). Using the GraphPad Prism.9.5 Software and Origin 2024 software was performed the statistical analysis. Statistical analysis between multiple groups used one-way analysis of variance (ANOVA) followed by Tukey post hoc test and two-sample *t*-test for the comparison between two samples.

## Funding

The authors would like to acknowledge the following funding which has provided the necessary financial support for this work. 10.13039/501100021171Basic and Applied Basic Research Foundation of Guangdong Province (2023A1515110332, 2025A1515010615, 2022A1515012460), Basic and Applied Basic Research Foundation of Guangzhou (2024A04J3464), The construction of high-level talents in high-level disciplines in high-level universities of 10.13039/501100010096Southern Medical University (22G601), 10.13039/501100002858China Postdoctoral Science Foundation (2021M703765), 10.13039/100016698National Natural Science Foundation of China (82072528), Natural Science Foundation project of Guangdong Provincial (2022A1515012460).

## Declaration of competing interest

The authors declare that they have no known competing financial interests or personal relationships that could have appeared to influence the work reported in this paper.

## Data Availability

Data will be made available on request.
