# Supplementary material for: Robust and ultra-stable nanohesive-based solid-like slippery coating under dynamic blood flow environment for durable prevention of thrombosis and biofouling
Source: Mater Today Bio. 2025 Oct 21;35:102449. doi: 10.1016/j.mtbio.2025.102449 (PMC12593591; doi:10.1016/j.mtbio.2025.102449)
Supplement: Multimedia component 1 [file mmc1.docx]

**Supporting Information for**

**Robust and Ultra-stable** **Nanohesive-Based Solid-Like Slippery Coating Under Dynamic Blood Flow Environment for Durable Prevention of Thrombosis and Biofouling**

Shu Zhang^1+^, Jihua Zou^1,2+^, Yupeng Xiao^1^, Xiaoying Qiu^1^, Yao Shen^1^, Yijin Zhao^1^, Tao Fan^1^, Manxu Zheng^1^, Guozhi Huang^1^*, Qing Zeng^1^*, Chengduan Yang^1^*

^1^Department of Rehabilitation Medicine, Zhujiang Hospital, Southern Medical University, School of Rehabilitation Sciences, Southern Medical University, Guangzhou, China

^2^Faculty of Health and Social Sciences, Hong Kong Polytechnic University, Kowloon, HongKong, SAR China

^+^These authors contributed equally to this work.

*To whom correspondence may be addressed. Corresponding to: Chengduan Yang, [yangchd13@smu.edu.cn;](mailto:yangchd13@smu.edu.cn;) Qing Zeng, [zengqingyang203@126.com;](mailto:zengqingyang203@126.com;) Guozhi [Huang, drhuang66@163.com;](mailto:Huang,drhuang66@163.com;)

**Methods**

**Preparation of “Solid-Like” Slippery Coating (SSC)**

At 25°C, 0.3 g of silica nanoparticles (anhydrous, 99.5%, Macklin) (~50 nm, ~15 nm, ~100 nm, ~300 nm), 10.0 g of silicone oil (viscosity 10 cSt, including -COOH terminated (customized by ZILIBON, DongGuan), -OH terminated (Sigma-Aldrich) and -OCH_3_ terminated (Sigma-Aldrich)), 10.0 g of ethyl acetate (analytical grade), 5.0 g of silane coupling agent (Shandong Yousuo Chemical Technology Co., Ltd.) were mixed and stirred at 500 rpm/min for 12 hours to obtain solution A; 5.0 g of epoxy resin (Shandong Yousuo Chemical Technology Co., Ltd.) was completely dissolved in 5.0 g of ethyl acetate as solution B. Solution A and solution B were mixed and stirred at 500 rpm/min for 1.0 hour to complete the chemical reaction. In addition, the ratio of nanoparticles and silicone oil was adjusted separately to obtain the other four SSC-COOH (0.03:100, 0.3:100, 30:100, 300:100) to investigate the effect of nanoparticle concentration on the structure and function of SSC-COOH.

The specific processing parameters of plasma technology (O_2_, TS-PL05, Tonson Tech) were as follows: All above samples were exposed to a vacuum environment of 80 Pa at a frequency of 40 kHz for 3 minutes. Then, the cleaned and plasma technology treated medical grade catheters (3 mm inner diameter, 4 mm outer diameter), glass plates (18 mm×18 mm×0.13 mm, BKMAM), copper (purity≥ 99%), aluminum (1060#, 35 mm×15 mm×2 mm, purity≥ 99.5%), silicon, polyisoprene (rubber) and polyethylene terephthalate (PET) are obtained by atomizing spray for 3 seconds at a distance of 15 cm (the solution concentration is 100%, the thickness is approximately 10 μm). Among them, SSC with thicknesses of 20, 50 and 100 μm were obtained respectively by extending the spraying time to 6, 15 and 30 seconds. Then removed and placed on a platform at the tilted angle of 30° for 1 hour to allow the excess coating to flow off and form a uniform and stable coating layer on the sample surfaces. Subsequent spontaneous drying at room temperature for 24 hours yielded a stable, solid and dry SSC. In this experiment, unless otherwise specified, we all used samples with a thickness of 10 μm as the research objects.

**Preparation of liquid-infused surfaces (LIS)**

After the primitive glass and primitive catheter were activated by plasma (The method was the same as above.), samples were immersed in a liquid silane solution (Trichloro-1H,1H,2H,2H-perfluorooctylsilane, TFOS, Sigma-Aldrich) for 12 hour at 80℃. Treated samples were rinsed with anhydrous ethanol and deionized water, and three times with pure ethanol. Rinsed samples were gently heated at 60 °C overnight at atmospheric pressure, and obtained the covalent grafted layer. Then, the mobile liquid perfluorocarbon layer is applied. The coated samples were dip-coated in perfluorodecalin (PFD, Sigma-Aldrich). Finally, the LIS modified surfaces were obtained.

**Preparation of liquid-infused surfaces (SLIPS)**

Glass substrate was selected for SLIPS modification. Before SLIPS modification, clean glass needs to be etched with hydrofluoric acid (HF) for 5 minutes (concentration 50%, Sigma-Aldrich). Then, they were immersed in DI water and saturated sodium bicarbonate solution (Aladdin) respectively to stop the reaction. After washing with a large amount of DI water, the porous surface was obtained after drying. The samples were then soaked in TFOS for 24 hours to carry out silanization treatment. Finally, PFD was added to the fluorinated surface, and the excess oil layer was removed after a short period of time to obtain a smooth SLIPS surface.

**Characterization of SSCMs**

After the SSC was formed, we used glass plates instead of medical catheters for oil-test paper and transparency test. First, UV-visible near-infrared spectroscopy (PE lambda 750) was used to measure the PG and SSC-COOH-MG transmittance ranging from 400 to 800 nm. Second, SSC modified glass (SSCMG) and unmodified primitive glass (PG) were placed over the logo and letters to compare the difference in optical transparency of the glass before and after modification. The SSC thickness was measured with a paint film-thickness gauge (Sanliang CT400, Japan). The chemical structure of the characteristic peak / bond type (including amino-SiO_2_ nanoparticles, -COOH terminated silicone oil, SSC-COOH solution, SSC-COOH and PC surfaces (control group)) was detected by Fourier infrared spectroscopy (FTIR) (Spectrum TWO, PerkinElmer). Then, oil-test papers were placed on the surface of SSC modified surfaces and lubricant-infused porous surfaces (SLIPS) to assess the dryness of the surfaces and to determine if there was any residual lubricant on the surfaces. Meanwhile, the surface morphology and smoothness of the catheters surface morphology before and after modification were observed by Scanning Electron Microscope (SEM) (HITACHI S-3000N). The changes in the contents of major elements on the surface of the medical tubing before and after modification were analyzed by X-ray photoelectron spectroscopy (XPS) (EscaLab 250Xi, Thermo), including the Si 2p peak, Si 2s peak, C 1s peak, N 1s peak and the O 1s peak, to further determine that the SSC were successfully coated on the surface of the catheters. The thermal stability of amininated nanoparticles, epoxy resin, carboxyl terminated silicone oil, methoxy terminated silicone oil, SSC-COOH, SSC-OH and SSC-OCH_3_ was determined by thermoweight analyzer (Netzsch TG 209).

To evaluate the lubricity characteristics of SSC, various water-based liquid droplets (deionized (DI) water, dulbecco's modified eagle medium containing 10% fetal bovine serum (DMEM + 10% FBS), the bacterial solution at a concentration of 1×(10^9^-10^10^)/ml in broth and whole blood) were measured for wetting phenomena on the SSC-COOH, SLIPS and LIS surfaces. Unmodified primitive glass (PG) surfaces were used as control groups. In the SSC-COOH and PG groups, test droplets were labeled with Rhodamine B (1 μg/ml). First, 2 μl droplets with different surface tensions (deionized (DI) water, dulbecco's modified eagle medium containing 10% fetal bovine serum (DMEM + 10% FBS), the bacterial solution at a concentration of 1×(10^9^-10^10^)/ml in broth and whole blood) were taken. Static contact angles (CAs) as a measure of surface wettability. Droplets were added to the SSC surface. Then CAs, Slide angles (SAs), Contact Angle hysteresis (CAH = *θ*_adv_ - *θ*_rec_), and droplet surface tension were measured using a contact angle meter (SDC-350H, China) to characterize the wetting properties of the SSC-COOH, LIS, SLIPS and PG surface. For the measurement of the surface free energy of solids, we tested the contact angle values of two droplets, DI water and ethanol, through the OWRK model in the contact angle measuring instrument program, and obtained the surface free energy of SSC-COOH through the model calculation. Also, use a pipette to take 10 μl droplets and observe the dynamic sliding of different droplets on different surfaces (tilt angle was 30°). In addition, the two protein dilutions were subjected to slip tests (fluorescein isothiocyanate bovine serum albumin (FITC-BSA, 5.12 mg/ml, Solarbio) and fluorescent fibrinogen (Fg, 5 mg/ml, Solarbio). After the sliding experiment was completed, the residual liquid droplets on the surface of the SSC-COOH and PG were observed with a fluorescence microscope.

Tape-peeling test: The tape-peeling test was conducted according to the ASTM D3359-17 standard. For the test, the coating surface was first mounted with a 3MTM VHB tape (width ~2 cm, adhesion value ~3000 N/m), and then was pressed by rolling over a copper rod (weight ~4 kg).

**The mechanism and performance of stability of SSCMs**

The shear force of the normal vessel wall was 1 ~ 7 Pa, the blood viscosity was 0.004 Pa·s, and the catheter diameter was 3 mm. The conversion process is as follows:

$\gamma=\frac{\tau}{\eta}$ (1)

$Q=\frac{\gamma\cdot d}{8}\cdot\pi r^{2}$ (2)

Where $\gamma$ is shear rate, τ is shear force, η is the blood viscosity, $Q$ is volume flow rate, d is catheter diameter and $r$ is catheter radius.

The samples were exposed to high physiological shear rate (up to 1750 s^-1^, the equivalent volume flow rate was about 280 ml/min).

The SSC modified glass prepared from different silicone oils (-COOH, -OH, -OCH_3_ terminated) were soaked in 1 M NaCl, CaCl_2_ and FeCl_3_ solution (blood simulator) at 1750 s^-1^ shear rate for 6 hours and 24 hours, using the same method measuring the DI water wetting and sliding property of the surface at the two time points.

When exploring the effect of hydrogen bonds on coating stability, the SSC-COOH, SSC-OH and SSC-OCH_3_ surfaces were soaked in neutral high-speed blood flow shocks (1750 s^-1^ shear rate for 3 days and 7 days) and measured the CAs, SA and CAH variation of water on the three surfaces after treatment.

Furthermore, the SSC solution was reprepared 50 nm-nanoparticles, without nanoparticles, 15 nm-nanoparticles, 100 nm-nanoparticles, 300 nm-nanoparticles and coated on the surface of catheters to prepare 50 nm SSC-COOH, non-nanoparticles SSC-COOH, 15 nm SSC-COOH, 100 nm SSC-COOH and 300 nm SSC-COOH groups. Meanwhile, these groups were used the same method measuring the DI water wetting property (including SA and CAH) of the surface before and after repeated blood rash impact wear (1750 s^-1^ shear rate for 3 days).

**Anti-adhesion Properties of SSCMC**

**Anti-protein adhesion test of SSCMC (fluorescein isothiocyanate bovine serum albumin (FITC-BSA), and fluorescent fibrinogen (Fg)).**

To investigate the anti-protein adhesion effect of SSC in the experiment, fluorescein isothiocyanate bovine serum albumin (FITC-BSA, the protein concentration was about 5.12 mg/ml, MW ~ 68 kDa, Solarbio) and fluorescent fibrinogen (Fg, the protein concentration was about 5 mg/ml, Solarbio) were selected as representative proteins, and the SSC-COOH group modified catheters (SSCMC) samples were placed in 96-well plates with the above two proteins for 24 hours of static incubation at 37 °C in the dark environment, while the unmodified PC samples were used as the control group. After incubation, the samples were rinsed once with PBS buffer (0.01 M, pH = 7.2) to remove the protein solution remaining on the surface. After the above treatments were completed, the adhesion of protein molecules on the surface of the two groups of samples under the same exposure time were recorded using a fluorescence microscope (Ti2-E, Nikon), and the relative fluorescence intensities of the adherent proteins on the surface of the two groups of samples were analyzed by quantitative statistics using Image J.

**Anti-bacteria adhesion test of SSCMC. (*Escherichia coli* (*E. coli*) and *Staphylococcus aureus* (*S. aureus*))**

To investigate the anti-bacterial adhesion effect of SSC in the experiment, *E. coli* (ATCC25922) and *S. aureus* (ATCC6538) (both purchased from Shanghai LuWei Microbial Tech. CO. LTD) were selected as representative bacteria. SSCMC samples were incubated with 100 μl of bacterial *E. coli* or *S. aureus* at a concentration of 1× (10^9^-10^10^)/ml (PBS) colony in 37℃ inside a 96-well plate for 24 hours and then stained with 10 μg/ml green fluorescent dye (SYTO-9, Thermo Fisher Scientific). After staining, bacteria were washed once with PBS, the surface adhesion of the two samples at the same exposure time was recorded with a fluorescence microscope, and the relative fluorescence intensity of the surface adhesion bacteria of the two samples was quantified by Image J.

**Anti-cells adhesion test of SSCMC. (Mouse embryonic fibroblasts (NIH 3T3) and mouse monocyte macrophage (RAW 264.7))**

To investigate the effect of anti-cell adhesion effect of SSC in the experiment, mouse embryonic fibroblasts NIH 3T3 and mouse monocyte macrophage RAW 264.7 were used as representative cells to evaluate the anti-cell adhesion ability *in vitro*. Two cells of 1× (10^5^-10^6^) cell/ml were cultured for 24 hours with 90% DEME, 9% FES (FBS), 1% antibiotic medium, 37℃, 95% humidity and 5% CO_2_. After incubation, 10 μg/ml green fluorescent dye (Calcein AM, Thermo Fisher Scientific) was performed. After staining, the samples were washed once with PBS, the cell adhesion on the surface of two samples at the same exposure time was recorded with a fluorescence microscope, and the number of fluorescent cells on the surface of both groups was quantified by Image J.

**Test of protein activity**

To explore the effect of SSC on protein molecular activity, SSCMC samples were incubated with a concentration of 0.1 mg/mL (PBS) glucose oxidase (GOD, Aladin) at 37℃ for 24 hours. After the incubation, the activity of the protein molecules was analyzed using the GOD activity detection kit (Solarbio). The blank treatment and heating treatment (90℃, 0.5 hour) were also used as control group.

**Test of bacteria activity**

To explore the experiment of the influence of SSC on bacterial activity, *E. coli* and *S.aureus* were incubated with SSCMC samples for 24 hours at 37℃. After the incubation, the bacteria were stained live dead with 10 μg/ml green fluorescent dye (SYTO-9, Thermo Fisher Scientific, for labeling live bacteria) and 10 μg/ml red fluorescent dye (Propidium iodide (PI), Thermo Fisher Scientific, for labeling dead bacteria). A blank 96-well plate bottom was also used as a blank control group. The results of bacterial viability on the surface of both samples at the same exposure time were recorded using a fluorescence microscope and quantified by counting the percentage of viable and dead bacteria.

**Test of cells activity**

NIH 3T3 and RAW 264.7 cells were incubated with 96-well plates, blank 96-well plates containing SSCMC samples for 24 h at 37℃. After incubation, cell viability was observed with 10 μg/ml green fluorescent dye (Calcein AM for labeling live cells) and 10 μg/ml red fluorescent dye (Propidium Iodide for labeling dead cells). Live and dead cells on the surface of the two samples at the same exposure times were recorded with a fluorescence microscope, and the number of live and dead cells on the surface of the two samples was quantified by Image J.

***In vitro* hemolysis test**

Blood compatibility of SSC was evaluated by New Zealand rabbit blood. Briefly, the blood was left at 4℃ for 30 min, centrifuged at 1000 rpm/min for 10 min and the supernatant was removed. Cells were subsequently repeatedly washed 2 to 3 times with 0.9% NaCl and resuspended to obtain a red blood cell suspension at a concentration of 5%. 100 ul of red cell suspension was added to 900 ul 0.9% NaCl solution containing MC samples (SSC coverage area about 2 cm^2^), DI Water (Positive Control), 0.9% NaCl solution (negative control) and incubated for 1 hour at 37℃. After centrifugation at 1000 rpm/min for 10 min, 100 ul of supernatant from each group were taken and transferred to a microplate reader to read the absorbance of the supernatant at 540 nm. Each experiment was repeated three times. The calculation formula of hemolysis rate: [(OD*_SSCMC_* - OD*_Negative Control_*) / (OD*_Positive Control_* - OD*_Negative_* *_Control_*)] × 100%.

**Test of antithrombosis properties *in vitro and vivo***

In order to prove that SSC can significantly prevent platelet adhesion in living life, effectively prevent thrombosis, and avoid the activation of various anti-inflammatory factors in the immune system *in vivo*, the New Zealand rabbits (common grade, male, 14-15 w, 3.0-3.5 kg) were used as living subjects for *in vivo* and external blood circulation experiments. Animal handling and surgical procedures *in vitro* and *in vivo* thrombophilia tests strictly followed the Chinese local ethics committee and experimental animal management rules. The animal experiment was approved by the laboratory animal welfare and ethics committee of the Zhu Jiang hospital of Southern Medical University (LAEC-2024-036FS, LAEC-2024-036FS2).

After 2 hours of extracorporeal circulation, the vessels were recovered and washed with normal saline, SEM was used to observe the platelet adhesion on the surface of the two groups of catheters was compared, and the catheter occlusion and blood flow rate were quantitatively analyzed using computer image analysis software.

A 0.5-meter long SSCMC was selected for simulated clinical application, mounted into a rabbit arteriovenous shunt circuit and with an unmodified PC as a control group. At predetermined time intervals (0, 5, 30, 60 min), rabbit blood was collected for biochemical analysis, including thrombin-antithrombin complex (TAT, CUSABIO), plasmin- α2 plasmin inhibitor complex (PIC, mlbio), thrombomodulin (TM, mlbio), platelet (PLT), leukocyte (WBC), serum albumin (ALB, proteintech), C reactive protein (CRP, proteintech), tumor necrosis factor-α (TNF-α, proteintech), inflammation and immunosuppression representative factors (IL-6, IL-10, proteintech), including coagulation, inflammation, and organ function. Furthermore, the potential toxicity of the materials and coatings to organs and tissues was further evaluated by measuring the concentration of plasma concentration of the liver enzyme alanine aminotransferase (ALT, CUSABIO) and the renal parameters serum creatinine (Scr, Beyotime).

The adsorption of FITC-Fg on SSC surface was determined by cardiopulmonary bypass. First, fresh rabbit blood was pumped into a vacuum tube containing sodium citrate (anticoagulant to blood volume ratio of 1 : 9) and labeled with FITC-Fg (5 v/v%). PC and SSCMC form closed-loop extracorporeal circulation system respectively. Then, PC and SSCMC form closed-loop extracorporeal circulation system respectively. After flowing for 1 hour (1 ml/min), the catheter was washed with PBS for 1 to 2 times, and the adsorption of Fg on the two surfaces was observed under fluorescence microscope. The anticoagulation ability of SSC was observed by *in vitro* coagulation reaction. 2 ml fresh blood was added to the ordinary centrifuge tube and the SSC modified centrifuge tube respectively, and the blood coagulation time was observed and recorded.

**The biosafety of SSCMC *in vivo***

In order to further evaluate the biocompatibility of SSC *in vivo*, SSCMC was implanted in the subcutaneous tissue of SD rats for 7 days to observe skin healing, acute inflammatory response and early foreign body reaction. Meanwhile, the original antibacterial catheter was used as a control group.

**Test of durability of SSCMC and LIS**

The stability and long-term resistance to biofouling properties of SSC-COOH. Firstly, SSC-COOH modified catheters samples (SSCMC) and LIS modified catheters samples were exposed to low physiological shear rate (up to 250 s^-1^, the equivalent volume flow rate was about 40 ml/min) in fluid environments (blood simulators, pH = 7.3, viscosity was about 4 mPa·s) using microfluidic devices (Kamoer) can be treated for 7, 14, 30 days, and for 7 days, respectively. after exposure to high physiological shear rate (up to 1750 s^-1^, the equivalent volume flow rate was about 280 ml/min).

The following treatments were performed: first, SSCMC and LIS samples were placed in 96-well plates with the two proteins (FITC-BSA and Fg) for 72 hours at 37℃ in the dark, respectively. While the unmodified PC group was used as the control group. After the incubation, rinse once with PBS buffer (0.01M, pH = 7.2) to remove the protein solution remaining on the surface. After completing the above treatments, the surface protein molecule adhesion of all samples at the same exposure time, and the relative fluorescence intensity of the surface adhesion proteins of all samples was analyzed by Image J quantitative statistics.

Then two bacteria (*E. coli* and *S. aureus*) with 100 ul of 3 × (10^8^-10^9^)/ml (PBS) colonies were incubated with the treated SSCMC and LIS samples at 37℃ for 72h, and the bacterial broth medium was replaced every 24 hours. After the incubation, bacterial staining was performed with 10 μg/ml SYTO-9. After staining, they were washed once with PBS and bacterial adhesion on the surface of both samples at the same exposure time was recorded by fluorescence microscope and relative fluorescence intensity of adhering bacteria on the surface of both samples was quantified by Image J.

Finally, NIH 3T3 and RAW 264.7 cells at concentrations of 4 × (10^4^-10^5^) cell/ml were incubated with the treated SSCMC and LIS samples in 96-well plates of cell culture medium with antibiotics for 72 hours. The cell culture medium was changed every 36 hours, and the incubation was 10 μg/ml CAM. After the staining, cells were washed once with PBS buffer, cell adhesion on the surface of both samples at the same exposure time was recorded by fluorescence microscopy, and the number of fluorescent cells on the surface of both samples was counted quantitatively by using Image J.

Similarly, in antithrombotic experiments, after the PBS treated SSCMC, LIS and unmodified PC were extracorporeal for 2 hours, intraductal thrombosis was observed and thrombus weight, occlusion rate and blood flow rate were determined.

In addition, the SSC-COOH samples were immersed in different physiological conditions (including pH = 5.5, pH = 7.5, 0.9% NaCl) for 30 days, the changes of anti-protein adhesion and slip properties were observed.

The vessel wall friction is usually 1 ~ 7 Pa and the friction area of the target catheter is 0.000942 cm^2^ (catheter diameter 3 mm, length 10 cm). The surface of SSC-COOH is repeatedly rubbed on 1500# sandpaper, applying a weight of about 1g (equivalent to 10 Pa of the vessel wall friction) for a cycle distance of 20 cm, and SA is measured once per 250 cycles.

The specific cross-cutting test operation is as follows: Ensure that the coating is fully cured and that the test area is clean, dry and smooth. Apply even force to smoothly press the gripper over the coating all the way to the substrate in one go. Make the second set of cuts perpendicular to the first set to form a 6 × 6 grid pattern, generating small squares. Gently brush and sweep the cutting area to remove any fallen coating fragments. Stick the pressure-sensitive tape onto the grid pattern, ensuring it is flat and in full contact. Smooth the tape hard with your fingers or an eraser. Within 90 seconds after pasting, grasp the free end of the tape and tear it off quickly (in a quick motion) at an Angle as close to 180° as possible. Then, conduct the DI water’s SA test. Set the LIS surface as the control group. Besides, the treatment was carried out under high pressure conditions of 0.1 MPa and 120℃ for 60 minutes respectively, and at an ultrasonic frequency of 20 kHz for 30 minutes. Systematically evaluate the changes in sliding performance before and after treatment.

**Supplementary Figures**


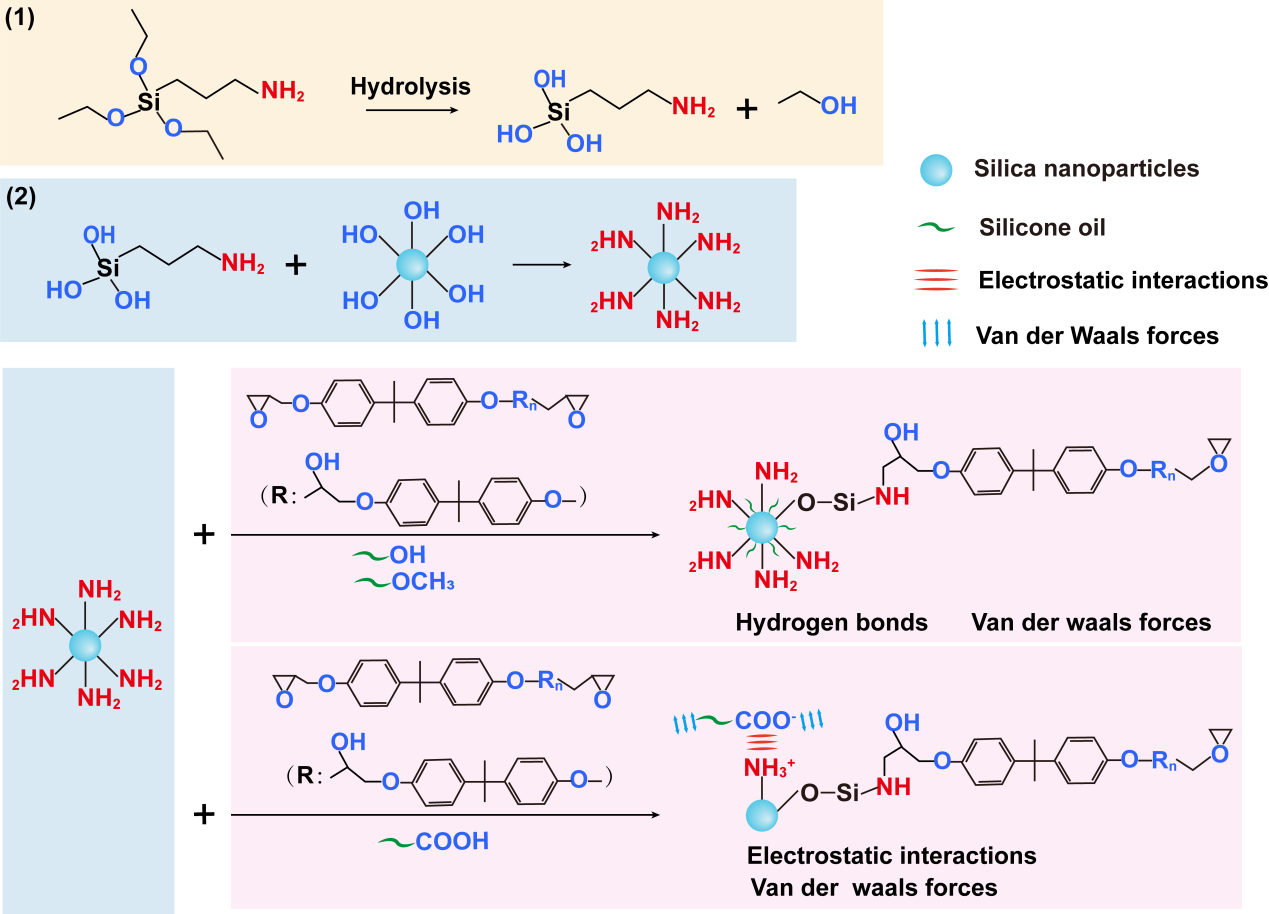


**Figure S1. Schematic of the reaction process of the SSC**. Process involving a series of hydrolysis-condensation-crosslinking reactions. First, the silane coupling agent hydrolyzes the at room temperature. Then condenses with the amino-functionalized SiO_2_ nanoparticles (amino-SiO_2_) nanoparticles to form a functionalized surface. At the same time, the silicone oil (-COOH terminated, -OH terminated and -OCH_3_ terminated) were trapped and stored on the rough structural surface of the silica nanoparticles. Finally, the nanoparticles are cross-linked with the epoxy resin, so that the oil storage silica nanoparticles are embedded inside and on the surface of the epoxy resin, and a translucent and smooth solid solution can be obtained.


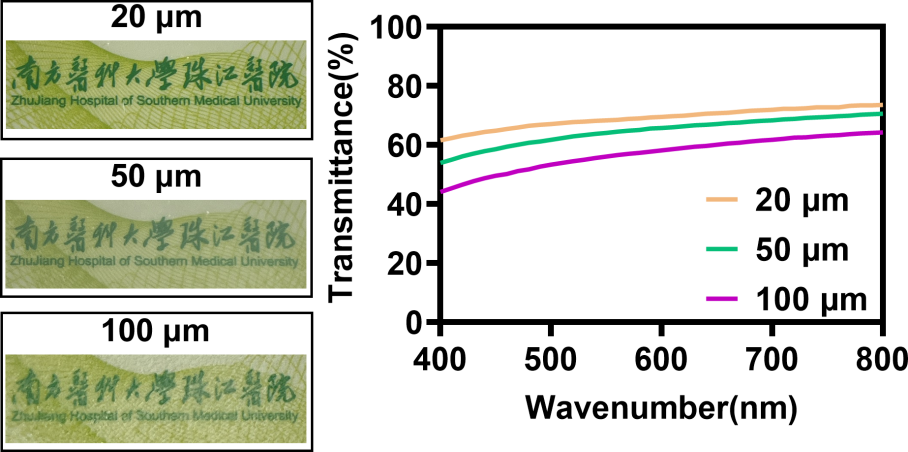


**Figure S2.** The influence of coating thickness on the transparency of glass. Different thicknesses can be obtained by adjusting the spraying time (6, 15, 30 seconds), and the specific thickness value can be obtained through a film thickness gauge (Sanliang CT400, Japan). The surface results showed that the thicker the coating, the lower the transparency.


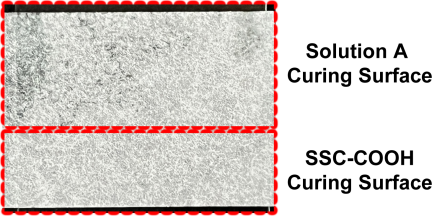


**Figure S3.** After the same spraying and curing treatment of the synthesis solution A of SSC and SSC-COOH solution, the surface was covered with oil test paper to observe some free lubricant. As can be seen from the figure, there is still a small amount of free lubricant in solution A. This also shows that epoxy resin achieves a “physical cage” effect to prevent the leakage of silicone oil not captured by nanoparticles.

.


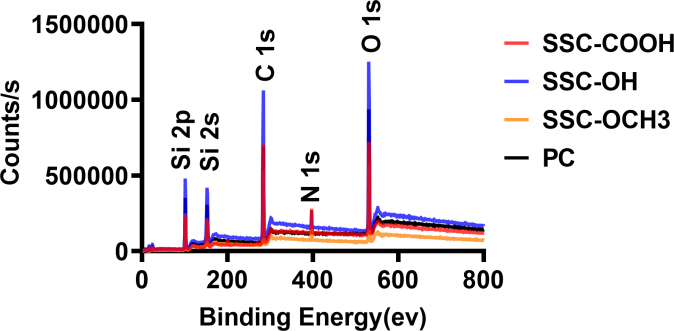


**Figure S4.** X-ray photoelectron spectroscopy (XPS) was used to analyze the elemental composition of SSCMC (including SSC-COOH, SSC-OH, SSC-OCH_3_), the primitive catheter served as a control group.


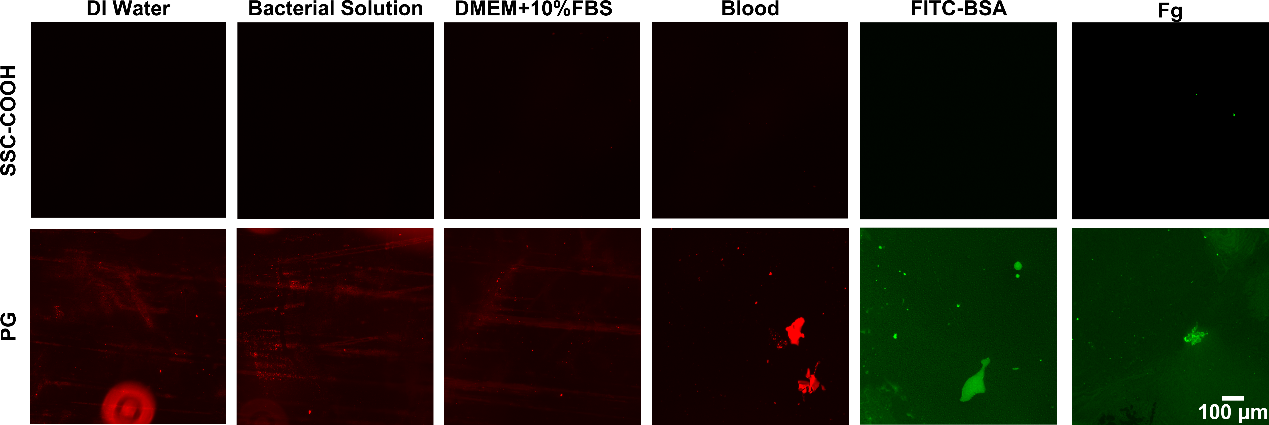


**Figure S5.** After the sliding experiment was completed, the residual liquid droplets on the surface of the SSC-COOH were observed with a fluorescence microscope and almost no red/green fluorescence was observed on the surface of SSC-COOH, indicating that there were no residual liquid imprinting and excellent “slippery” performance. On the contrary, the surface of PG contains residual fluorescent liquid to varying degrees.


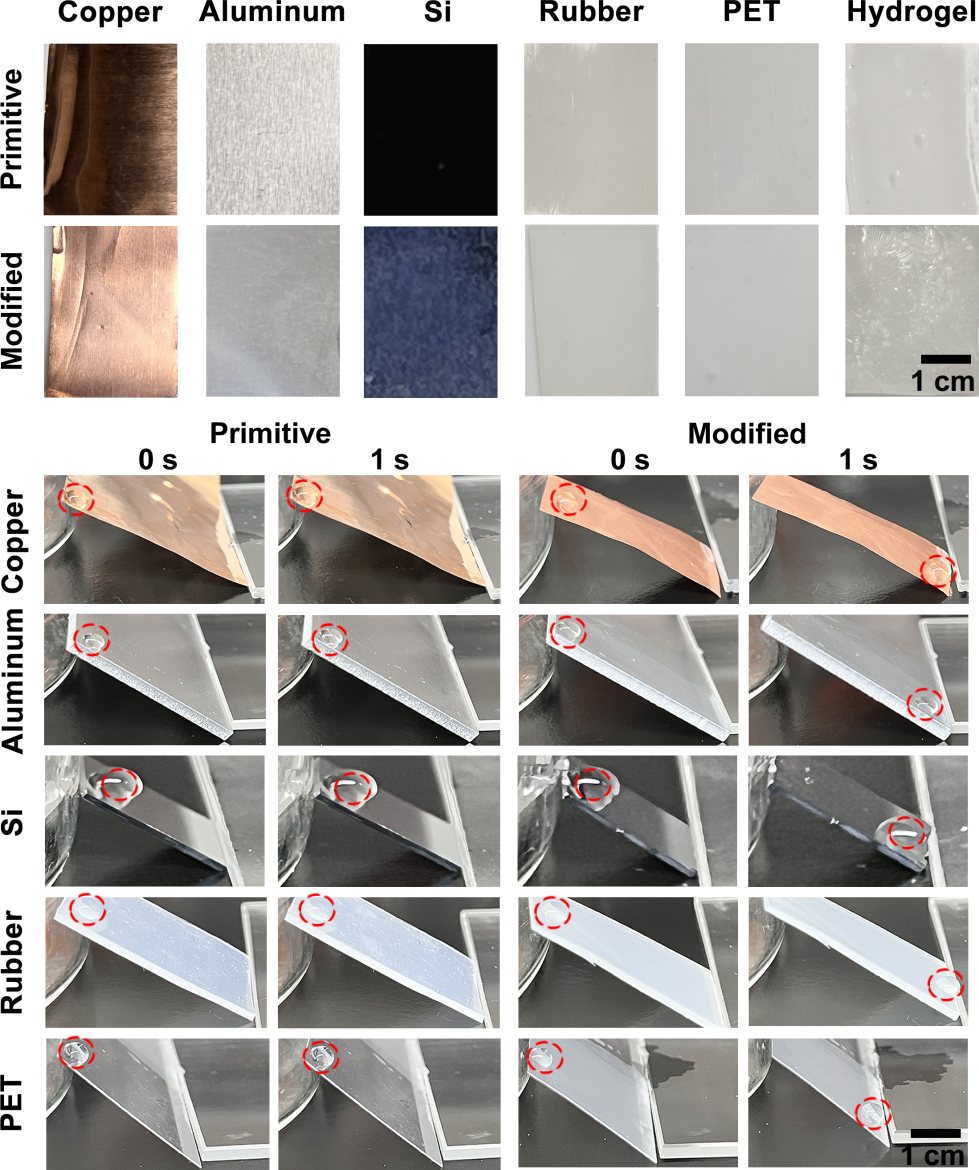


**Figure S6. Comparison of lubrication performance before and after modifying SSC with different substrate surfaces.** After SSC surface modification, these surfaces can promote water slip without any residue (the tilted angle was 30°, within 1 second), showing excellent slippery properties. This suggested that the "solid-like" slippery coating has the potential feasibility of conferring various blood-contacting biomedical materials to inhibit thrombosis and biological contamination.

**
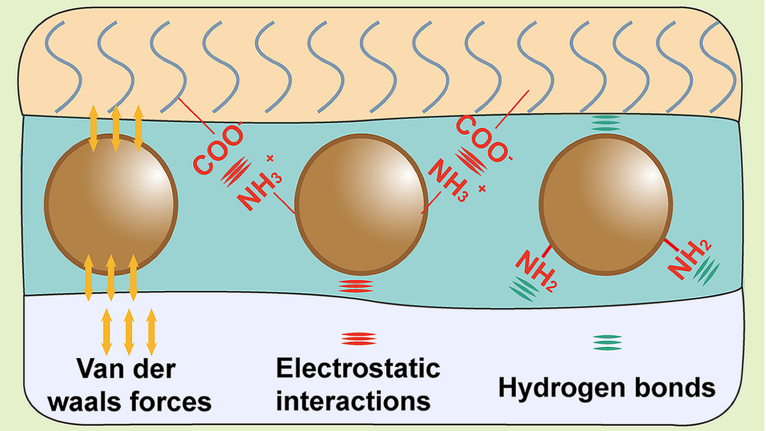
**

**Figure S7.** Various interaction forces formed between nanoparticles and the interfaces.

**
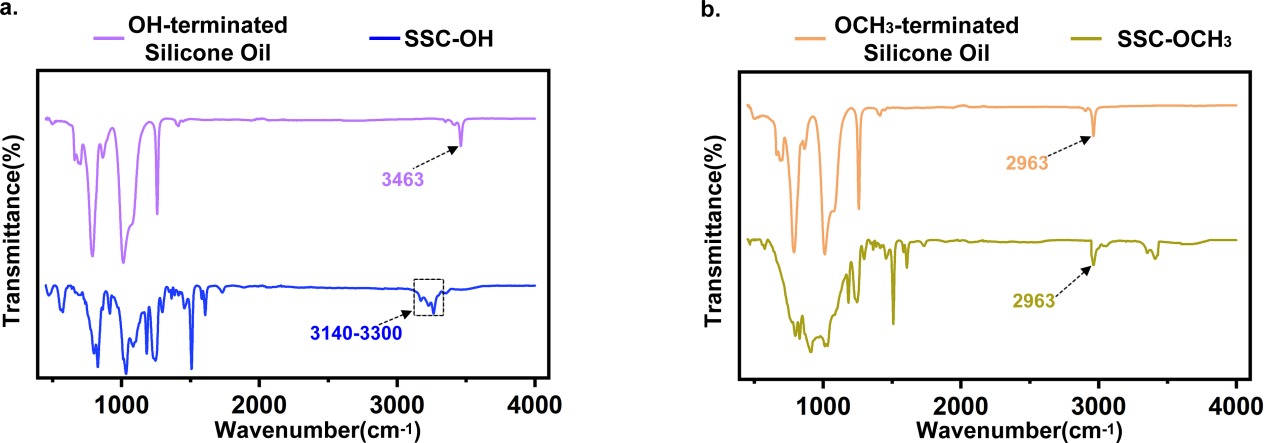
**

**Figure S8. Results of the FTIR spectrum analyses of hydroxy-terminated silicone oil and SSC-OH, methoxy-terminated silicone oil and SSC-OCH_3_ surface.** SSC-OH and SSC-OCH_3_ were formed by hydroxy (-OH) terminated silicone oil and methoxy (-OCH_3_) terminated silicone oil instead of -COOH terminated silicone oil, respectively. For the SSC-OH (Figure S8a), The O-H expansion vibration absorption peak (sharp peak) in the silicon oil was distributed in the range of about 3463 cm^-1^. In the SSC-OH system, the O-H expansion vibration absorption peak (wide peak) shifted to the lower wave number range (3140 ~ 3300 cm^-1^). These changes mean that there was a hydrogen bond formation between the nanoparticle and OH-terminated silicone oil in the SSC-OH system, and its stability source depends on the action of the hydrogen bond. However, in SSC-OCH_3_ (Figure S8b), the C-H peak from silicone oil remained at 2963 cm^-1^ before and after the reaction. The absorption peaks of the related groups did not move towards the low wave number in the SSC-OCH_3_ system, which means that the related hydrogen bonds are not formed in SSC-OCH_3_.


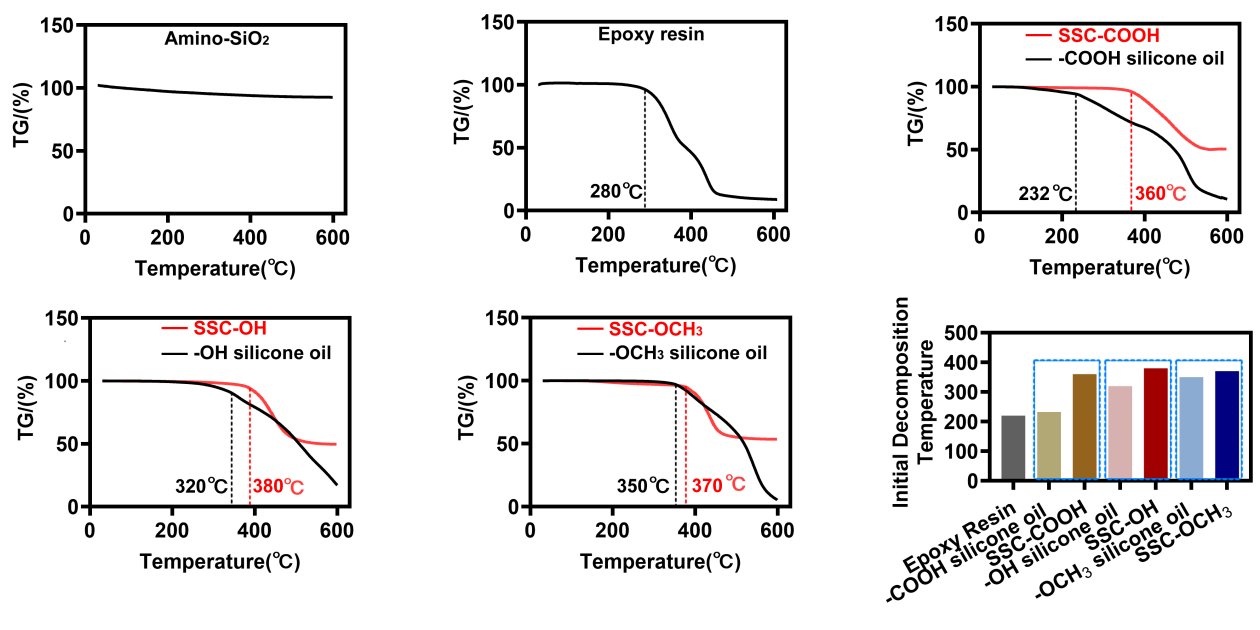


**Figure S9. Thermogravimetric analysis results.** The stability effects of electrostatic interactions and hydrogen bonds on SSC were further analyzed from the perspective of thermal stability.


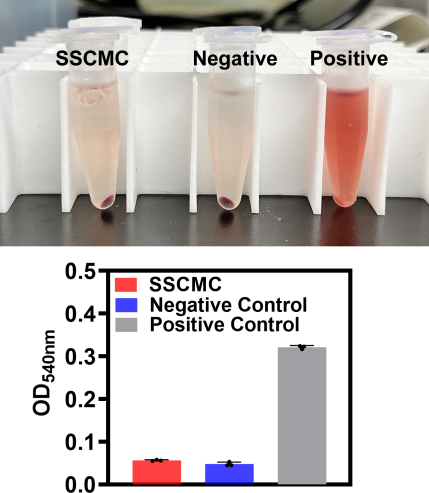


**Figure S10. SSCMC *in vitro* hemolysis assay.** After incubating the samples with the red blood cell suspension for 1 hour, the hemolysis rate in the SSCMC group was 3.15% (<5% indicates good blood compatibility).


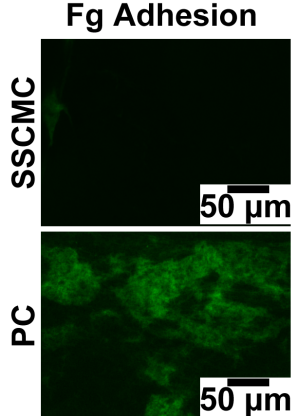


**Figure S11. Fibrinogen adhesion.** Fibrin adherence was observed after fresh rabbit blood was labeled with Fg and cardiopulmonary bypass was performed for 1 hour.


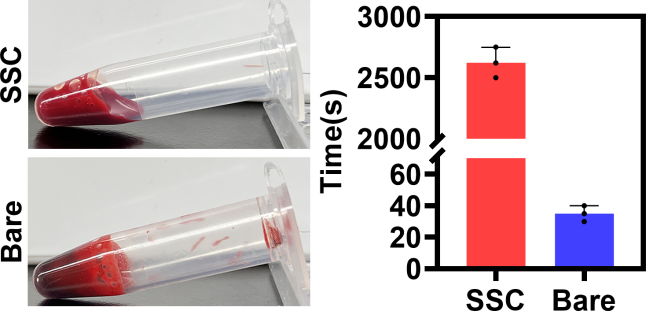


**Figure S12. *In vitro* coagulation reaction**. Optical image of blood coagulation and statistics of coagulation time. Error bar represents the mean ± SD. Significance was calculated by one-way analysis of variance. N = 3, averaged.


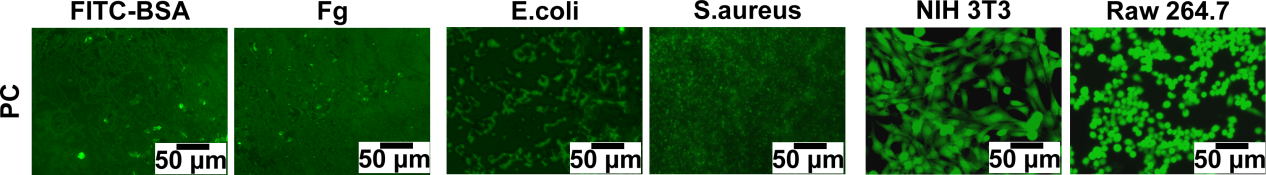


**Figure S13. Long-term anti-adhesion test of PC group.** Representative fluorescence pictures of unmodified PC catheters after incubation with protein (FITC-BSA and Fg), bacteria (*E. coli* and *S. aureus*), cells (NIH 3T3 and RAW 264.7) for 3 days.


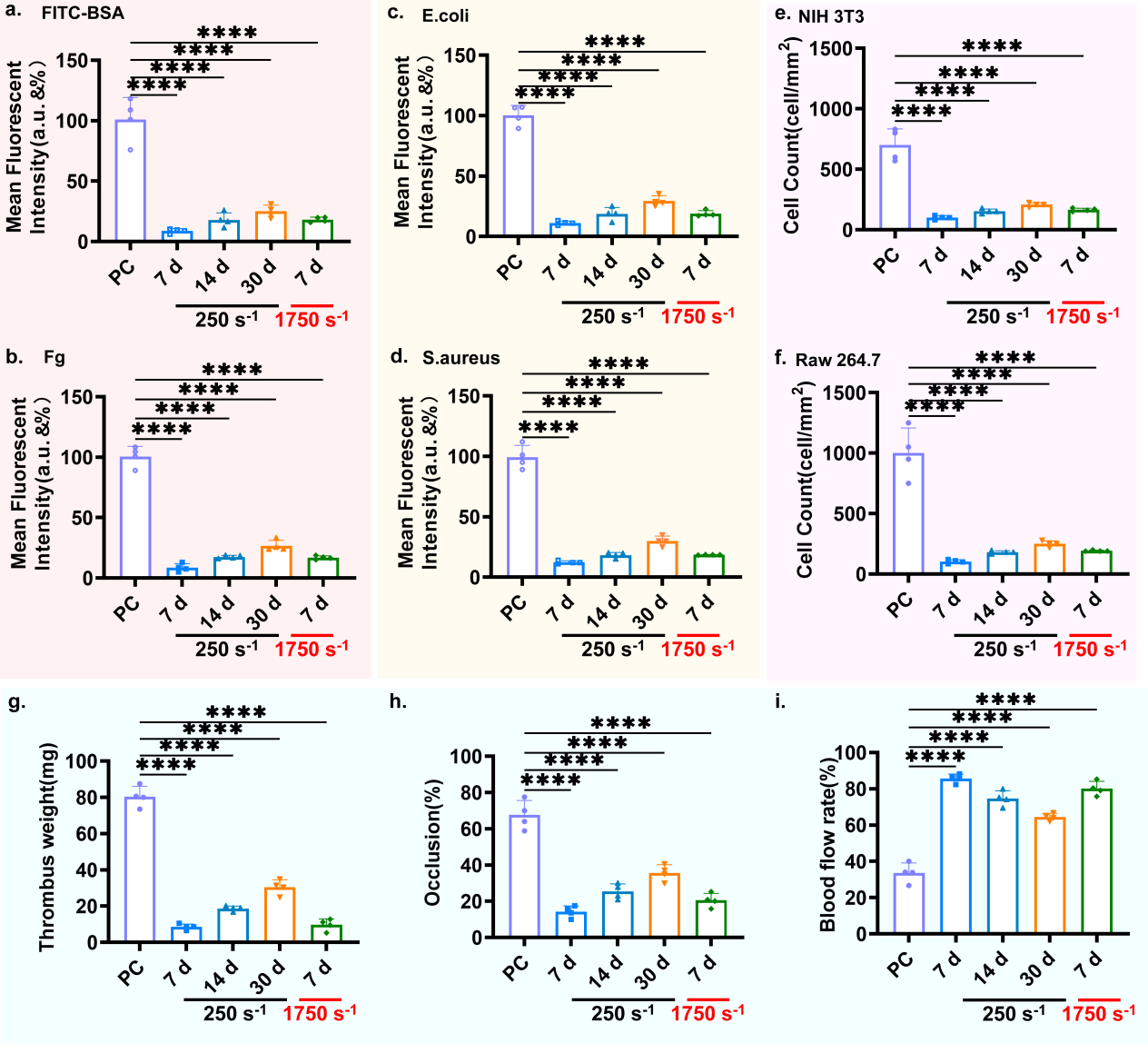


**Figure S14. Test of the durability of SSCMC.** a) ~ f) The adhesion results of proteins, bacteria and cells of the unmodified PC group and the SSCMC group after the various treatments mentioned above were quantitatively analyzed, and there were significant differences. g) ~ i) The weight of thrombus formed in the inner lumen of the catheters, occlusion, and blood flow rate of each group were quantified, and significant differences were found. Error bar represents the mean ± SD. Significance was calculated by one-way analysis of variance. **p* < 0.05, ***p* < 0.01, ****p* < 0.01, *****p* < 0.0001, ns : not significant. N = 4, averaged.


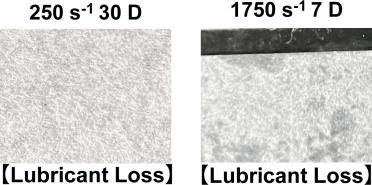


**Figure S15. Comparison of** **lubrication layer loss on LIS.** After simulated blood flow low shear rate environment treatment (250 s^-1^ for 7, 14, 30 days and high shear rate environment impact (1750 s^-1^ for 7 days), respectively, the lubrication layer on LIS surfaces was almost lost. This also means that the lubricating fluid layers was unable to withstand the fluid impact of a long time or high shear environment, and the anti-adhesion properties were basically lost.

**
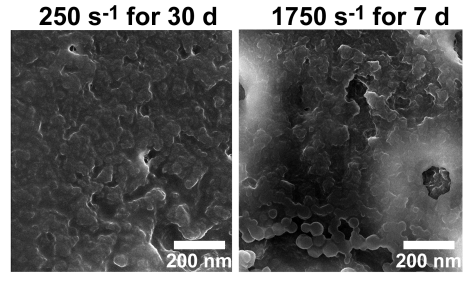
**

**Figure S16. The changes of SSCMC cross section were observed by SEM after the two fluids were impinged (~ 250 s^-1^ for 30 days, ~ 1750 s^-1^ for 7 days).** The results showed that the cross section was not damaged and still maintained a dense structure.

**
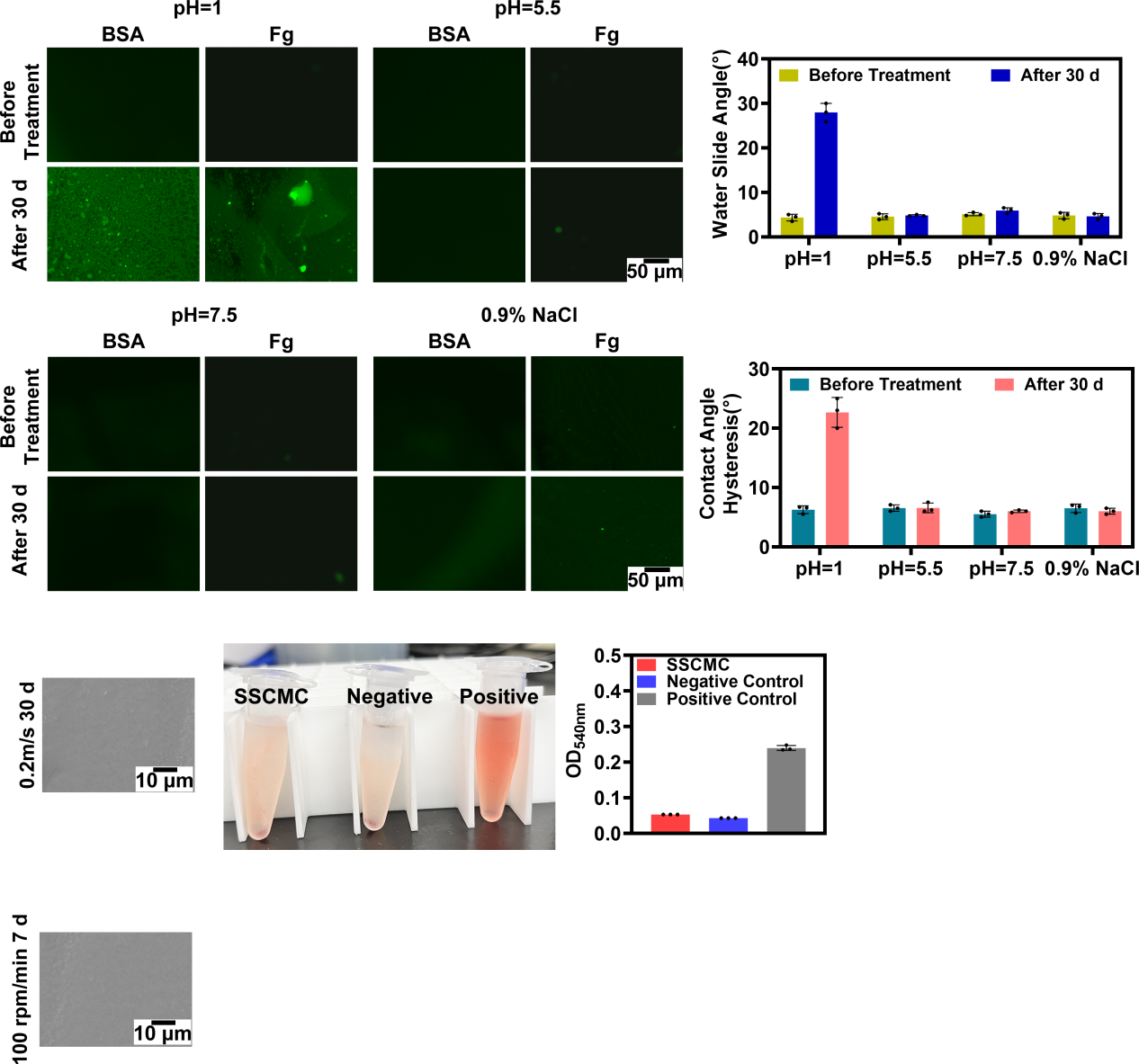
**

**Figure S17. Life expectancy results of SSCMs under different physiological conditions.** The slippery properties and anti-adhesion properties of the SSC-COOH surfaces did not change significantly under nearly neutral physiological conditions (pH = 5.5, pH = 7.5, 0.9% NaCl). In contrast, in the strongly acidic environment, its slippery properties and anti-adhesion properties decrease sharply, which may be that the strongly acidic environment breaks the electrostatic interactions of the coating.


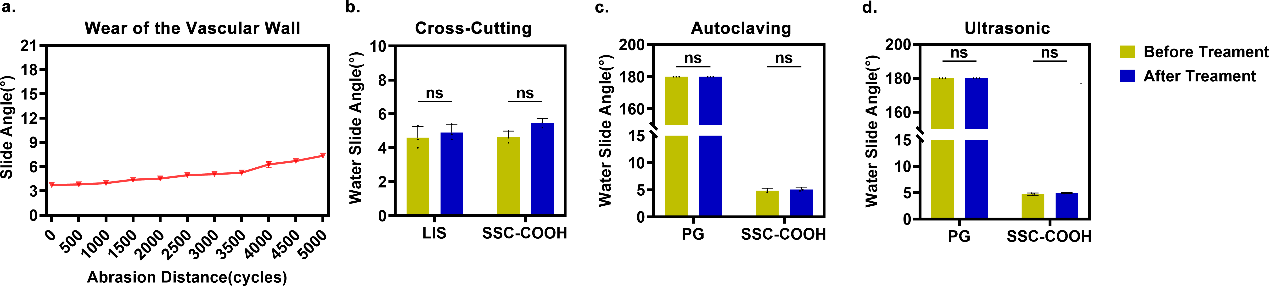


**Figure S18. Sanded paper wear simulates normal vessel wall friction to verify SSCMs wear resistance.** a) The friction of blood vessel walls was simulated through 5,000 cycles of sandpaper wear, b) cross-cutting test, c) autoclaving test (120℃, 0.1 MPa) treatment for 60 minutes and d) ultrasonic treatment for 30 minutes, the SA of SSC-COOH was still less than 10° after above treatments, maintaining an excellent sliding property, indicating that SSC-COOH could effectively resist normal vascular wall friction and had excellent long-term wear resistance.
